# Localization-limited exciton oscillator strength in colloidal CdSe nanoplatelets revealed by the optically induced stark effect

**DOI:** 10.1038/s41377-021-00548-z

**Published:** 2021-05-31

**Authors:** Pieter Geiregat, Carmelita Rodá, Ivo Tanghe, Shalini Singh, Alessio Di Giacomo, Delphine Lebrun, Gianluca Grimaldi, Jorick Maes, Dries Van Thourhout, Iwan Moreels, Arjan J. Houtepen, Zeger Hens

**Affiliations:** 1 Physics and Chemistry of Nanostructures, Department of Chemistry, Ghent University, Gent, Belgium; 2 Center for Nano and Biophotonics, Ghent University, Gent, Belgium; 3 Photonics Research Group, Ghent University, Gent, Belgium; 4 Department of Chemical Sciences and Bernal Institute, University of Limerick, Limerick, Ireland; 5 Center for Nanophotonics, NWO-Institute AMOLF, Science Park 104, 1098 XG Amsterdam, The Netherlands; 6 Opto-Electronic Materials Section, Department of Chemical Engineering, Delft University, Delft, The Netherlands

**Keywords:** Optical spectroscopy, Quantum dots, Nanoparticles

## Abstract

2D materials are considered for applications that require strong light-matter interaction because of the apparently giant oscillator strength of the exciton transitions in the absorbance spectrum. Nevertheless, the effective oscillator strengths of these transitions have been scarcely reported, nor is there a consistent interpretation of the obtained values. Here, we analyse the transition dipole moment and the ensuing oscillator strength of the exciton transition in 2D CdSe nanoplatelets by means of the optically induced Stark effect (OSE). Intriguingly, we find that the exciton absorption line reacts to a high intensity optical field as a transition with an oscillator strength *F*_*Stark*_ that is 50 times smaller than expected based on the linear absorption coefficient. We propose that the pronounced exciton absorption line should be seen as the sum of multiple, low oscillator strength transitions, rather than a single high oscillator strength one, a feat we assign to strong exciton center-of-mass localization. Within the quantum mechanical description of excitons, this 50-fold difference between both oscillator strengths corresponds to the ratio between the coherence area of the exciton’s center of mass and the total area, which yields a coherence area of a mere 6.1 nm^2^. Since we find that the coherence area increases with reducing temperature, we conclude that thermal effects, related to lattice vibrations, contribute to exciton localization. In further support of this localization model, we show that *F*_*Stark*_ is independent of the nanoplatelet area, correctly predicts the radiative lifetime, and lines up for strongly confined quantum dot systems.

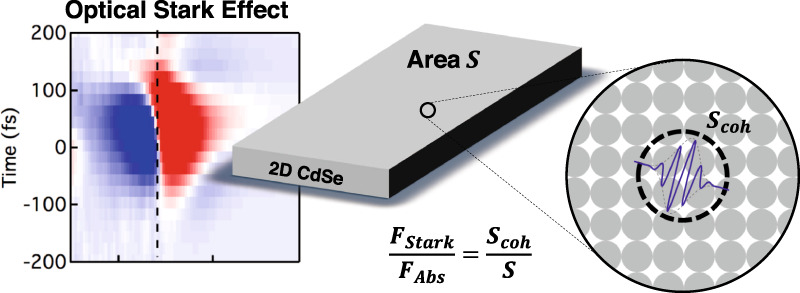

## Introduction

Colloidal quantum wells of CdSe^1,2^ have attracted much attention in the past years due to narrow, exciton-related absorption features, an increased light-matter interaction, strong light amplification^3–7^ and exciton-polariton formation^8,9^. As two-dimensional (2D) materials, these so-called nanoplatelets fall in between atomically thin 2D materials, such as transition metal di-chalcogenides^10,11^, and the usually much thicker epitaxially grown quantum wells. Moreover, being capped by organic ligands, nanoplatelets are intrinsically embedded within a low permittivity environment. This dielectric confinement substantially enhances the exciton binding energy^12^. While the exciton binding energy of 15 meV in bulk CdSe should increase to 60 meV in a 2D CdSe quantum well^13^, typical estimates amount to ~190 meV for 4.5 monolayer (1.21 nm) thick CdSe nanoplatelets^3,7,14,15^. With such binding energies, excitons in nanoplatelets are stable quasi-particles at room temperature, and exciton-related transitions have been used to develop room temperature nanoplatelet-based light emitting diodes^16^ and lasers^3^.

At cryogenic temperatures, the heavy-hole bright exciton in CdSe nanoplatelets was found to exhibit a radiative decay rate of ~1 ps^−1^, a rate that also determined the exciton dephasing^14^. Similar observations were made in the case of epitaxial quantum wells^17,18^, and attributed to the large in-plane coherence area of the exciton center-of-mass motion in these systems. Intriguingly, recent reports based on state-filling models proposed that even at room temperature, this coherence might be close to 100 nm^2^, a number that seems incompatible with the nanosecond radiative lifetime reported by various authors^19–21^. In addition, several studies indicated the potential for strong coupling of excitonic transitions with the light field at room temperature using 4.5 monolayer CdSe nanoplatelets, a feat that requires narrow transition lines with large oscillator strength^8,9^. Using an elaborate fitting procedure of exciton-polariton dispersion curves, heavy hole transition dipole moments of 575 Debye (D) at room temperature were extracted. Although promising, such dipole moments seem disruptively large as compared to literature reports on comparable material systems, such as epitaxial quantum wells (6 D)^22^, three and two-dimensional perovskites (46 and 15 D, respectively)^23,24^, carbon nanotubes (12 D)^25^, and transition metal-dichalcogenides (7 D for WSe_2_^26^, 51 D for WS_2_^27^, and 9 D for MoSe_2_ at 77K)^28^.

In studies, the optical Stark effect (OSE) is used as a method to extract the desired dipole moment^22,27^. Using OSE spectroscopy, one pumps the material using a femtosecond pump pulse detuned relative to the exciton transition and measures the induced energy shift of the exciton using a broad, white-light probe pulse. This method alleviates the need for electrical contacting^29^ and does not rely on real charge carriers, thereby eliminating any spurious effects of defect trapping and assumptions on state-filling or electron-hole overlap^19,20^. Recent work by Diroll showed that also CdSe nanoplatelets display such a Stark effect and dipole moments in the range 15–23 D were extracted, numbers which are very much in line with other 2D materials^30^. However, translating such dipole moments into dimensionless oscillator strengths leads to numbers of around one. Since oscillator strengths of 5–15 are routinely found for 0D colloidal quantum dots, such a result questions whether light-matter coupling in 2D nanoplatelets is particularly strong and warrants a deeper investigation into the exciton oscillator strength of these materials.

In this work, we extend the use of polarization resolved OSE spectroscopy to develop a consistent interpretation of the oscillator strength of 2D excitons. First, we confirm the report of Diroll, measuring transition dipole moments of 18 D for 4.5 ML CdSe nanoplatelets. Next, we translate these values into a dimensionless oscillator strength and conclude it is fifty times smaller than the oscillator strength of the exciton transition as derived from the linear absorbance spectrum using previously published methods^31^. We argue that this discrepancy results from a strong localization of the center-of-mass of the exciton. Since a nanoplatelet can host multiple localized excitons, center-of-mass localization can strongly reduce the oscillator strength of a single exciton transition—as measured by the Stark-effect—without affecting the overall oscillator strength of the exciton absorption. Translating this interpretation into a quantum mechanical description, we show that the ratio between the oscillator strengths yields the coherence area of the heavy-hole bright exciton at room temperature, resulting in a value of ≈6.1 nm^2^. Interestingly, similar measurements at 77 K yield a coherence area of ≈12 nm^2^; a result suggesting that thermal effects such as lattice vibrations contribute significantly to exciton localization. Further supporting the interpretation of exciton localization, we show that the coherence area and the ensuing oscillator strength does not depend on the nanoplatelet area at room temperature, and that localization accounts for the radiative lifetime of the exciton. Finally, we show that the integrated band-edge absorbance yield similar oscillator strengths as the OSE experiment in the case of CdSe colloidal QDs, suggesting that such 0D systems effectively host electron-hole pairs delocalized over the entire nanocrystal volume at room temperature.

## Results

### The optical stark effect

**Fig. 1:**
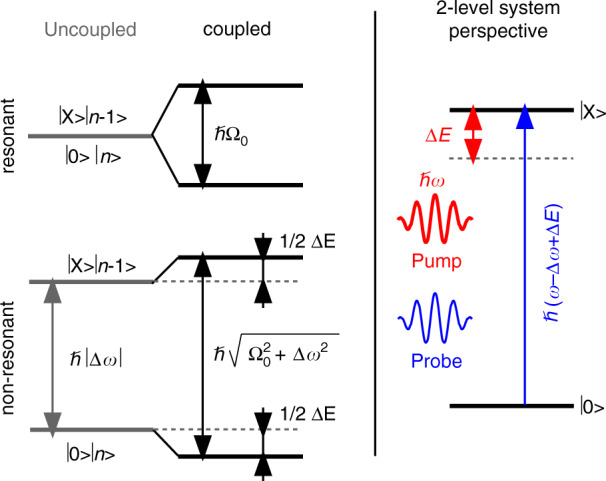
Scheme outlining the optical Stark effect. Coupling of a two-level system with a resonant photon field splits the degenerate states $\left|\ 0\ \right\rangle \left|\ n\ \right\rangle$ and $\left|\ {\rm{X}}\ \right\rangle \left|\ n-1\ \right\rangle$ by the Rabi frequency Ω_0_. For non-resonant optical fields, the effect is reduced to a mere shift of the states $\left|\ 0\ \right\rangle \left|\ n\ \right\rangle$ and $\left|\ {\rm{X}}\ \right\rangle \left|\ n-1\ \right\rangle$. In the case of negative detuning, this results in an increase Δ*E* of the transition energy between the two-level ground state $\left|\ 0\ \right\rangle$ and the two-level excited state $\left|\ {\rm{X}}\ \right\rangle$; a shift known as the optical Stark effect

To study the light-matter coupling in CdSe platelets, we assessed the optical Stark effect (OSE) of the heavy-hole exciton using white light pump-probe spectroscopy. All following optical experiments were carried out at room temperature, except when mentioned otherwiseThe OSE is typically described within a dressed atom picture as the result of the coherent interaction between a two-level system and a photon field. Figure 1 shows the principle behind the OSE applied to a two-level system that corresponds to a platelet in its ground state $\left|\ 0\ \right\rangle$ and in the state $\left|\ {\rm{X}}\ \right\rangle$ where it holds a single exciton. In that case, a resonant pump at frequency *ω* = *ω*_0→X_ will couple the degenerate states $\left|\ 0\ \right\rangle \left|\ n\ \right\rangle$ and $\left|\ {\rm{X}}\ \right\rangle \left|\ n-1\ \right\rangle$ – in which the platelet is either in the ground state $\left|\ 0\ \right\rangle$ or the excited state $\left|\ {\rm{X}}\ \right\rangle$ and the optical field contains *n* or *n* − 1 photons – to form mixed light-matter states split by the Rabi frequency ${{{\Omega }}}_{0}^{2}={{\mathcal{E}}}^{2}{\mu }_{0\to {\rm{X}}}^{2}/{\hbar }^{2}$. Here, ${\mathcal{E}}$ is the root mean square of the electric field associated with the pump light and *μ*_0→X_ is the transition dipole moment of the ground state to exciton transition.

**Fig. 2:**
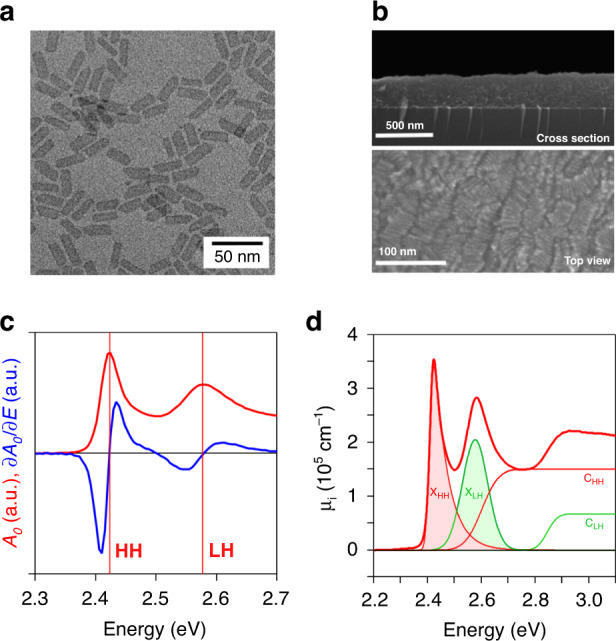
Sample overview. **a** Transmission electron microscope image of 4.5 monolayer thick CdSe platelets with a lateral area of 34 × 9.6 nm^2^. **b** Scanning electron microscope cross section of 288 ± 30 nm thick film of CdSe platelets showing clear out-of-plane stacking. **c** Linear absorption spectrum A_0_ of platelets in thin film (black) and its first derivative $-\frac{\partial {A}_{0}}{\partial E}$ (red). **d** Decomposition of the absorption spectrum, here represented as an intrinsic absorption coefficient^31^, into excitonic (X) and 2D continuum (C) contributions for light (LH, green) and heavy (HH, red) related transitions, see main text

When the pump laser is off resonance, the uncoupled states $\left|\ 0\ \right\rangle \left|\ n\ \right\rangle$ and $\left|\ {\rm{X}}\ \right\rangle \left|\ n-1\ \right\rangle$ are split by the absolute value of the laser detuning Δ*ω* = *ω* − *ω*_0→X_, see Fig. 2a. In that case, coupling only leads to a mere shift of these initial states when the laser detuning strongly exceeds the Rabi frequency. For a negative detuning (*ω* < *ω*_0→X_), the case shown in Fig. 1, the result is an increased splitting between the lower energy state $\left|\ 0\ \right\rangle \left|\ n\ \right\rangle$ and the higher energy state $\left|\ X\ \right\rangle \left|\ n-1\ \right\rangle$. From the perspective of the original two-level system, this leads to an increase of the transition energy between the states $\left|\ 0\ \right\rangle$ and $\left|\ {\rm{X}}\ \right\rangle$, i.e., the optical Stark effect, by an amount Δ*E* given by (see Fig. 2a):

1 $${{\Delta }}E=\hbar \sqrt{{{{\Omega }}}_{0}^{2}+{{\Delta }}{\omega }^{2}}-\hbar | {{\Delta }}\omega | \approx \frac{{\mu }_{0\to {\rm{X}}}^{2}{{\mathcal{E}}}^{2}}{\hbar | {{\Delta }}\omega | }$$

Negative detuning has the advantage that the pump pulse can induce an OSE, without creating real excitons by 1-photon absorption. Furthermore, we ensured that pump intensities were sufficiently low so as to avoid significant 2-photon absorption^32^. Under such conditions, a coincident white light probe beam can measure the shift of the exciton absorbance proper, without state-filling, exciton saturation or band-gap renormalization obscuring the measured transient absorption spectrum; a process represented in Fig. 1^33,34^.

In practice, we analyzed the optically induced Stark effect by illuminating a film of CdSe nanoplatelets using a 120 fs pump pulse detuned to lower photon energies as compared to the heavy hole exciton transition, see “Methods” section for details. The transient shift of the exciton transition induced by the pump was then analyzed by means of a broadband probe, of which we measured the change in absorbance Δ*A* = *A* − *A*_0_ as a function of the pump-probe delay. This delay was varied via a mechanical delay stage to obtain 2D time-energy maps of Δ*A* for a given detuning of the pump laser. Moreover, as CdSe nanoplatelets are not isotropic, we used two different combinations of linear polarization for the pump and probe, denoted here as co-polarized (*xx*) and cross-polarized (*xy*).

### The optical stark effect of CdSe nanoplatelets

In this study, we worked with 4.5 monolayer (ML) CdSe nanoplatelets, which were synthesized using a modification of the procedure proposed by Ithurria et al. (see “Methods” Section)^1^. Figure 2a displays a typical bright field transmission electron microscope (TEM) image of the nanoplatelets^2^. From the TEM image, we obtained the average lateral dimensions of the nanoplatelets. Unless stated otherwise, results reported in this manuscript pertain to a sample with a 34 × 9.6 nm^2^ area, see Supplementary Information S1. For the optical experiments, thin films of CdSe nanoplatelets were deposited on a transparent fused silica substrate using spincoating from *n*-heptane, forming ~250 nm thick smooth films, see Fig. 2b. We preferred thin films in this case to avoid strong, solvent-induced artifacts in the pump-probe measurements at short time delays that can occur when using apolar solvents, such as hexane.

Figure 2c shows the absorption spectrum and its first derivative of the nanoplatelets studied here as a function of energy. We observe pronounced features at 2.42 and 2.58 eV, related to the formation of heavy and light hole excitons, respectively^1^. We decomposed the absorption spectrum into contributions from the two exciton transitions and the associated 2D free carrier absorption profiles *C*_*HH*,*LH*_, see Supplementary Information S2 and Fig. 2d. A binding energy for the heavy hole exciton of ~190 meV is extracted, which matches well with literature estimates^14,29^. Importantly, such a binding energy corresponds to a 2D Bohr radius of 1.5 nm. Note that this Bohr radius is substantially smaller than the lateral extension of the nanoplatelets, which implies that excitons only exhibit weak lateral confinement. The fluorescence decay of these samples with 65% quantum yield reveals an average lifetime of 6.6 ns, as is shown in Supplementary Information S3.

**Fig. 3:**
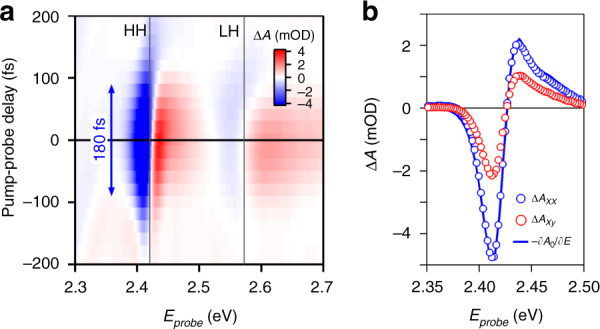
Pump-probe spectroscopy. **a** Color map of the differential absorbance ΔA as function of the probe energy (horizontal axis) and the pump-probe delay (vertical axis) measured on a film of 4.5 ML CdSe nanoplatelets using a 580 nm (2.14 eV) pump and a copolarized pump and probe, measured at room temperature. The lines labeled HH and LH indicate the position of the heavy and light-hole resonances, respectively. The 180 fs arrow outlines the full width at half maximum of the absorption transient. **b** Extracted Δ*A* spectrum for zero time delay at 0.4 GW/cm^2^ for (blue markers, *xx*) co- and (red markers, *xy*) cross-polarized pump and probe signals, together with (full blue line) the rescaled first derivative of the linear absorption spectrum ∂*A*_0_/∂*E*

Figure 3a shows a typical 2D map of Δ*A* recorded on a film of 4.5 ML CdSe nanoplatelets using a pump pulse at 580 nm (2.14 eV, Δ = 288 meV) for a parallel pump-probe polarization. One clearly observes distinct and short-lived anti-symmetric features around the HH and LH resonances. Looking at the transient absorbance spectrum at zero time delay (see Fig. 3b), we retrieve similar spectra for both combinations of pump-probe polarization, albeit with a different absolute signal for the same pump power. As highlighted in the case of Δ*A*_*xx*_, these spectra closely resemble the first derivative of the linear absorption spectrum. In that case, the reduced absorbance at the low energy side and the increased absorbance at the high energy side of the exciton absorption point toward a blueshift of the exciton transition, which is indeed what the optical Stark effect should bring about. Finally, Fig. 3a highlights the temporal width of the transient absorbance Δ*A* around the heavy-hole exciton. With a full width at half maximum of 180 fs, a number closely corresponding to the convolution of a 120 fs pump and a 120 fs probe, this implies that we indeed look at an instantaneous variation of the exciton absorbance. This agrees with the expected instantaneous nature of the coherent optical Stark effect since dephasing at room temperature was shown to be limited to sub-100 fs using 2D electronic spectroscopy^35,36^. Moreover, the lack of a long-lived bleach or photo-induced absorption confirms that the detuned pump laser used here does not create real excitons, see Supplementary Information S4. Given the agreement between the transient absorbance spectrum and the first derivative of the linear absorbance shown in Fig. 3b, we calculate the shift of the exciton transition as the coefficient relating Δ*A* and ∂*A*_0_/∂*E*:^37^

2 $${{\Delta }}A(E)=-\frac{\partial {A}_{0}(E)}{\partial E}\times {{\Delta }}E$$

A complication in the case of the anisotropic nanoplatelets studied here is that both the pump and probe polarization, and the orientation of the platelet relative to both, will influence the relation between the energy shift Δ*E* and the change in absorbance Δ*A*. In Supplementary Information S5, we explicitly consider the different combinations of nanoplatelet orientations and pump and probe polarizations to obtain expressions that relate Δ*A*_*xx*_ and Δ*A*_*xy*_ to the spectral shift Δ*E*:

3 $$\begin{array}{l}{{\Delta }}{A}_{xx}=-{f}_{xx}\frac{\partial {A}_{0}}{\partial E}\times {{\Delta }}E\\ {{\Delta }}{A}_{xy}=-{f}_{xy}\frac{\partial {A}_{0}}{\partial E}\times {{\Delta }}E\end{array}$$

The coefficients *f*_*ij*_ can be calculated when the dielectric parameters of the platelet environment are known. Taking the well-known scenario of a dilute dispersion of platelets in hexane, the correction coefficients *f*_*xx*_ and *f*_*xy*_ would amount to 0.73 and 0.35, respectively, see Supplementary Information S5. Figure 3b confirms that the co-polarized transient absorbance Δ*A*_*xx*_ is about twice as large as the cross-polarized transient absorbance Δ*A*_*xy*_. While this rough estimate agrees by-and-large with the calculated estimate of *f*_*xx*_/*f*_*xy*_ = 2.09, one should realize that the correction factors will be somewhat different for nanoplatelets in the thin films used here, due to the reduced dielectric screening, a point we will come back to later.

**Fig. 4:**
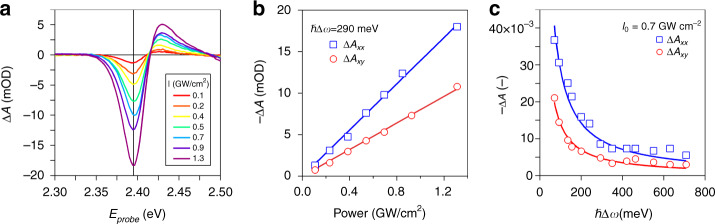
Fluence and detuning dependence. Room-temperature OSE spectroscopy (**a**) ΔA spectra at zero time delay for different pump intensities (in GW/cm^2^) and xx pump-probe polarizations. **b** Extracted energy shift *δE* (in meV) at 2.39 eV for increasing pump power, relative to *I*_0_ = 0.54 GW/cm^2^, and a fixed detuning of 288 meV. **c** Similar to (**b**) for a fixed pump intensity of 2.2 GW/cm^2^ and increased detuning Δ. Both in (**b**) and (**c**) the parallel *xx* (blue) and orthogonal *xy* (red) combinations of pump and probe are shown

Before quantifying the exciton shift Δ*E*, we first evaluated our results in view of Eq. (1) by analyzing Δ*A*_*xx*_ and Δ*A*_*xy*_ as a function of light intensity and detuning. As shown in Fig. 4a, changing the pump power at constant detuning Δ*ω* results in a strong increase in the transient absorbance at a given probe photon energy *E*_*probe*_. Figure 4b represents the variation of Δ*A*_*xx*_ and Δ*A*_*xy*_ at *E*_*probe*_ = 2.395 eV, which corresponds to the minimum of the Δ*A* spectrum as indicated in Fig. 4a. One readily sees that the transient absorbance scales linearly with the pump power, or with the electric field squared, a trend that agrees with the expression of the optical Stark effect and the linear relation between the transient absorbance and the shift Δ*E* of the exciton absorbance. Moreover, keeping the pump power constant and increasing the detuning ∣Δ*ω*∣ leads to a gradual reduction of Δ*A*_*xx*_ and Δ*A*_*xy*_ that scales as 1/∣Δ*ω*∣. We thus conclude that the transient absorbance does reflect the optical Stark effect of the exciton transition.

Finally, the ratio of the slopes of the best fits to Δ*A*_*xx*_ and Δ*A*_*xy*_ in Fig. 4b amounts to 1.75. This figure is somewhat smaller than the ratio of 2.09 we calculated for an apolar solvent environment, which probably reflects the reduced screening in the nanoplatelet film. In fact, a ratio *f*_*xx*_/*f*_*xy*_ = 1.75 would be obtained if the environment had a refractive index of 2.13, see Supplementary Information S5. This number is reasonable for a film consisting of close packed nanoplatelets capped by oleate ligands, and as such, we can reliably quantify Δ*E* using the corresponding correction coefficient *f*_*xx*_ = 0.75, see Supplementary Information S5.

### The oscillator strength of the exciton transition

Having validated the functional form set forth in Eq. (1) and having verified the conversion of Δ*A* into Δ*E*, we can use Eq. (1) to calculate the transition dipole moment *μ*_0→X_ linked to the formation of the bright heavy hole exciton. This yields a value of 18 D (see Supplementary Information S6), a figure that compares reasonably well to the 9 D found for slightly less confined 5.5 ML CdSe nanoplatelets using time-resolved AC Stark spectroscopy^29^. In addition, from the transition dipole moment, we can calculate the oscillator strength *F*_*Stark*_ of the exciton transition as^31^:

4 $${F}_{Stark}=\frac{2{m}_{e}\omega }{3{\hslash} {e}^{2}}\times {\mu}_{0\to {\rm{X}}}^{2}$$

Here, *m*_*e*_ is the free electron mass and *ω* is the angular frequency at the HH position. This expression yields *F*_*Stark*_ = 3.1 for the 4.5 ML CdSe platelets of 326 nm^2^, a number of the same magnitude as the oscillator strength *F* = 0.5−1 reported for the exciton transition in dielectrically confined single-layer perovskite nanoplatelets^24^. Interestingly, since transition energies are comparable for both systems, this points toward a stronger transition dipole moment in CdSe nanoplatelets. On the other hand, this oscillator strength is about 3–4 times smaller than the oscillator strength of the band-edge transition in CdSe QDs, for which values up to 10 were reported^38^. Clearly, this outcome challenges the idea of a giant oscillator strength of the exciton transition in room temperature CdSe nanoplatelets. We note that this conclusion also holds for all the reports in literature on OSE extracted dipole moments of varying 2D materials.

An alternative approach to determine the oscillator strength of the exciton transition starts from the linear absorption spectrum. When rescaling this spectrum to an intrinsic absorption coefficient spectrum *μ*_*i*_(*ℏω*), the oscillator strength of the exciton transition can be calculated from the integrated exciton absorption feature *μ*_*i*,*int*_, as outlined by the shaded area in Fig. 2d and Supplementary Information S2^31^:

5 $${F}_{Abs}=\frac{2{V}_{plat}{\epsilon }_{0}{n}_{s}c{m}_{e}}{e\pi \hbar | {f}_{LF}{| }^{2}}{\mu }_{i,int}^{-1}$$

Using an average local field factor of ∣*f*_*LF*_∣^2^ = 0.328 for the nanoplatelets in hexane used here, we estimate *F*_*Abs*_ = 165 ± 1.5 for the 4.5 ML platelets, see Supplementary Information S2. Intriguingly, this number exceeds the value obtained through the optical Stark effect by over a factor of 50, and it also strongly exceeds the oscillator strength reported for the band-edge transition of CdSe QDs^38^.

## Discussion

### Localized versus delocalized 2D excitons

**Fig. 5:**
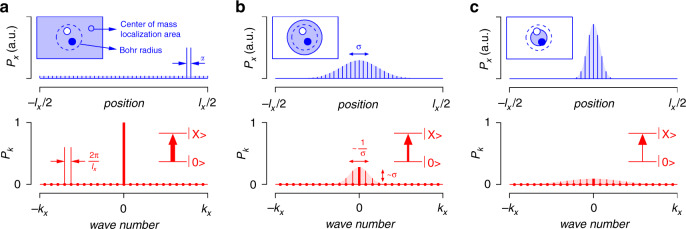
Theoretical model of localization. **a** Representation of the fully delocalized center-of-mass exciton state $\left|{\bf{K}}=0\right\rangle$ in (top) real space and (bottom) reciprocal space. The pictures highlight that this state is fully delocalized in real space with a position-independent probability and fully localized at **K** = 0 in reciprocal space. The inset top-left depict (filled blue area) the exciton center-of-mass (de)localization and (dashed line) the electron-hole separation. The inset bottom-right represents the optical transition from the nanoplatelet ground state to the $\left|{\bf{K}}=0\right\rangle$ state, where the thickness of the arrow is a measure of the oscillator strength. **b**–**c** The progressive localization of the exciton center of mass in real space leads to a progressive delocalization of the state in reciprocal space. As the contribution of the $\left|{\bf{K}}=0\right\rangle$ state is reduced, the oscillator strength of the optical transition from the ground state to a localized exciton goes down

To understand the very disparate oscillator strengths of the exciton transition as obtained from the optical Stark effect and the linear absorption spectrum, we start from the description of the 2D exciton wave function $\left|\ X\ \right\rangle$ as the product of a center-of-mass part $\left|\psi ({\bf{R}})\right\rangle$ and an internal part $\left|\chi ({\bf{r}})\right\rangle$. Here, **R** is the 2D position vector of the exciton center-of-mass, whereas **r** is the internal coordinate vector, measuring the difference between the position of the electron and the hole. The center-of-mass part $\left|\psi \right\rangle$ can be expanded in terms of plain waves, each characterized by a different 2D center-of-mass wavevector **K**. Of the different plain waves center-of-mass states, only the state $\left|{\bf{K}}=0\right\rangle$ is optically bright since the momentum change of the electronic states upon absorption of a photon is negligible. Importantly, the oscillator strength *F*_**K**=0_ of the transition from the ground state to this $\left|{\bf{K}}=0\right\rangle$ scales proportional to the platelet area, see Supporting Information S7^39^. As sketched in Figure 5a, the $\left|{\bf{K}}=0\right\rangle$ state describes in real space an exciton state with a center-of-mass that is fully delocalized across the entire nanoplatelet.

The delocalized exciton is an eigenstate of the periodic crystal lattice. Deviations from this perfect structure, such as (static) stacking faults of the crystal, thickness variations or changes in surface composition, and (dynamic) lattice vibrations, will tend to localize the exciton center-of-mass^40,41^. Such localized excitons can be described by wave packets, i.e., linear combinations of plain waves centered around a given center-of-mass wavevector. While a more general approach is presented in Supporting Information S7, we assess the effect of center-of-mass localization here by describing the center-of-mass wavefunction as a 2D Gaussian wave packet characterized in real space by the wave function *ψ*(**R**) and in reciprocal space by the corresponding Fourier transform *ϕ*(**K**):

6 $$\begin{array}{l}\psi ({\bf{R}})=\frac{1}{\sqrt{2\pi }\sigma }\exp \left(-\frac{{{\bf{R}}}^{2}}{4{\sigma }^{2}}\right)\\ \phi ({\bf{K}})=\sqrt{\frac{2}{\pi }}\sigma \exp \left(-{\sigma }^{2}{{\bf{K}}}^{2}\right)\end{array}$$

Here, *σ* measures the real-space delocalization of the center-of-mass (see Fig. 5). We should note that various localization mechanisms could give rise to different localized wavefunctions. However, as argued in Supporting Information S7, the conceptual picture put forward here does not depend on the assumption of a Gaussian wave packet.

As outlined in Fig. 5, increasing the localization in real space enhances the delocalization in reciprocal space, and reduces the contribution ∣*ϕ*(**0**)∣^2^ of the bright $\left|{\bf{K}}=0\right\rangle$ state to the localized exciton. Using this model of a Gaussian wave packet, the probability *P*_0_ to find a wave packet in the $\left|{\bf{K}}=0\right\rangle$ state can be written more precisely as:

7 $${P}_{0}={\left|{{\Phi }}({\bf{0}})\right|}^{2}\frac{4{\pi }^{2}}{S}=\frac{8\pi {\sigma }^{2}}{S}$$

Here, we used the fact that a single state occupies an area 4*π*^2^/*S* in reciprocal space, with *S* the platelet area (see Fig. 5 for a 1D equivalent). As a result, we find that the oscillator strength *F*_X_ to form a localized exciton state centered around **K** = 0 in reciprocal space can be written as a function of *F*_**K**=0_ as:

8 $${F}_{{\rm{X}}}=\frac{8\pi {\sigma }^{2}}{S}{F}_{{\bf{K}} = 0}=\frac{{S}_{coh}}{S}{F}_{{\bf{K}} = 0}$$

In the second equation, we interpreted 8*πσ*^2^ as the so-called coherence area *S*_*coh*_ of the wave packet, see Supporting Information S7. We thus retrieve the result already put forward by Feldmann et al.^42^, that exciton localization reduces the effective oscillator strength to form a 2D exciton by a factor *S*_*coh*_/*S*.

According to Eq. (8), a transition to form a localized exciton will have a smaller oscillator strength than the formation of the **K** = 0 exciton. However, each nanoplatelet will have multiple localized excitons as eigenstates, which are related with the plain wave basis by a unitary transformation. Therefore, the sum of the weight *P*_0_ (see Eq. (7)) over the different localized exciton states equals 1. We thus conclude that the distinction between localized and delocalized excitons does not affect the integrated absorption coefficient, such that *F*_*abs*_ yields an estimate of *F*_**K**=0_. On the other hand, when the coherence between these different localized exciton states addressed by the pump pulse is lost within the duration of that pulse, the optical Stark measurement addresses a collection of independent, localized excitons, and measures the transition dipole moment of forming a single localized exciton. Under such conditions, the ratio *F*_*Stark*_/*F*_*abs*_ provides a measure of the coherence area of the 2D exciton in CdSe nanoplatelets:

9 $$\frac{{F}_{Stark}}{{F}_{abs}}=\frac{{S}_{coh}}{S}$$

Note that a similar argument follows from Dicke’s treatment of optical transition in a collection of *n* two-level systems^43^, which would be localized excitons for the case studied in this work. Using the experimental values found for *F*_*Stark*_ and *F*_*abs*_ for the 326 nm^2^ sample, Eq. (9) yields a coherence area *S*_*coh*_ = 6.1 nm^2^. Returning to the Gaussian wavepacket, this corresponds to a radial spread on the exciton center-of-mass of ${\sigma }_{R}=\sqrt{2}\sigma \approx 0.70\ {\rm{nm}}$.

**Fig. 6:**
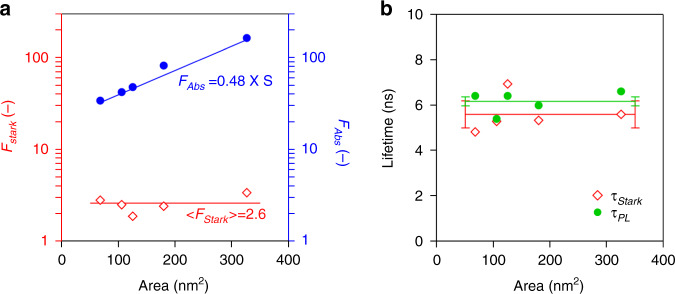
Connection between the oscillator strength, fluorescence lifetime and NPL area. **a** Plot showing the (red diamonds) measured room-temperature oscillator strength *F*_Stark_ and (blue circles) calculated *F*_Abs_ for different samples of 4.5 ML nanoplatelets with different surface areas. The horizontal red line represents the average oscillator strength <*F*_*Stark*_>, while the blue line displays a best fit of *F*_*Abs*_ to a straight line through the origin. Here, *S* is the nanoplatelet surface area in nm^2^. **b** Comparision of (red diamonds) room temperature radiative lifetime *τ*_*Stark*_ calculated from *F*_*Stark*_ and (green circles) the average luminescent lifetime *τ*_*PL*_ determined from luminescence decay measurements. The red and green line represent the sample average, whereas the error bars indicated the 95% confidence interval on both the averages

In line with the discussion of exciton absorption by Elliot^39^*, F*_*Abs*_ should increase proportionally with the nanoplatelet area *S*. This point is confirmed in Fig. 6a, where we display *F*_*Abs*_ as determined for 5 different sets of 4.5 ML nanoplatelets, with areas ranging from 68 to 326 nm^2^. The coherence area *S*_*coh*_, on the other hand, is determined by disturbances of the periodic crystal structure, such as lattice vibrations, impurities or an irregular surface termination (see later). Since the smallest area of the nanoplatelets studied still exceeds the estimated coherence area by one order of magnitude, we expect that such disturbances will yield the same coherence area – and thus the same *F*_*Stark*_ – for the different nanoplatelets studied. Fig. 6a confirms this point. While *F*_*Abs*_ increases almost 5-fold when raising the nanoplatelet area from 68 to 326 nm^2^, we obtain an average < *F*_*Stark*_ > of 2.6 without any systematic variation throughout the series of nanoplatelets analysed.

### The radiative lifetime of the exciton

Following the interpretation put forward in the previous section that *F*_*Stark*_ measures the oscillator strength of forming a single, localized exciton in a CdSe nanoplatelet, the same oscillator strength should determine the radiative recombination rate *k*_*rad*_ of such a localized exciton. Due to the small energy splitting between the dark and bright exciton states, the heavy hole exciton will only be half of the time in a bright state. Taking this occupation probability into account, *k*_*rad*_ can be obtained from *F*_*Stark*_ according to:^31,44^

10 $${k}_{rad}=\frac{{e}^{2}}{2\pi {\epsilon }_{0}{c}^{3}{m}_{e}}{n}_{s}| {f}_{LF}{| }^{2}{\omega }^{2}\frac{{F}_{Stark}}{2}$$

Here, all symbols have their usual meaning. Using *F*_*Stark*_ = 3.1 for the 326 nm^2^ sample, we obtain *k*_*rad*_ = 0.18 ns^−1^, a rate that corresponds to a radiative lifetime *τ*_*Stark*_ = 5.6 ns. This figure agrees well with the average luminescent lifetime *τ*_*PL*_ = 6.6 ns we determined from the fluorescence decay and is on par with the 6.4 ns measured by Morgan et al. for similar 4.5 ML CdSe nanoplatelets^20^. This lifetime is slightly larger than the 3.7 ns measured for highly efficient CdSe nanoplatelets passivated with a CdS crown by Leemans et al.^21^. Figure 6b compares the lifetime determined by OSE spectroscopy, *τ*_*Stark*_, and the luminescent lifetime, *τ*_*PL*_, for 4.5 ML CdSe nanoplatelets with various surface area. Similar to *F*_*Stark*_, we find that *τ*_*PL*_ is independent of the surface area and that the both numbers coincide within the statistical error on the analysis.

Based on this result, we conclude that interpreting the ratio *F*_*Stark*_/*F*_*Abs*_ as the ratio between the exciton coherence area and the total nanoplatelet area leads to a consistent interpretation. *F*_*Abs*_ is proportional to the total platelet area and determines the pronounced exciton feature in the absorption spectrum, while *F*_*Stark*_ is proportional to the coherence area of the localized exciton and is the relevant quantity to understand the radiative lifetime of the exciton. This result questions previously published estimates of the exciton area at room temperature of 96 nm^2^ or 21 nm^2^ for similar 4.5 ML nanoplatelets^19,20^. Such large coherence areas would yield significantly shorter room temperature radiative lifetimes than measured experimentally. Opposite from the approach used here, these estimates analysed the reduction of the exciton absorption with increasing exciton population from a state-filling perspective, a method hampered by accurate understanding of the saturation of exciton absorpiton in 2D systems, which are bosons showing no exclusion principle, and/or complications due to charge trapping. We stress that the OSE produces an oscillator strength that directly predicts the correct radiative lifetime, without need for corrections such as electron-hole overlap or thermal equilibria with supposed higher lying energy levels^20^.

### On the origin of exciton localization

While nanoplatelets appear as highly crystalline structures with a well-defined surface chemistry^2,45^, stacking faults or local variations in surface termination seem unavoidable in such extended crystallites. In addition, lattice vibrations make that atoms permanently oscillate around their equilibrium position. The resulting static and dynamic deviations of the actual electronic potential from that of the perfectly periodic crystal lattice localizes the exciton center-of-mass^40^. It was argued by Efros et al. that the exciton coherence area is related to the energy variations these deviations bring about^40^. This point can be understood from the inverse relation between localization in real space and delocalization in reciprocal space; a fundamental aspect of the uncertainty principle highlighted in Fig. 5. More quantitatively, to reduce the variation of the center-of-mass in real space to *σ*_*X*_, a variation of ${\sigma }_{{K}_{X}}\ge 1/2{\sigma }_{X}$ is needed at least. Reaching states with such a wave vector requires an additional energy Δ*E* relative to the **K** = 0 state of:

11 $${{\Delta }}E=\frac{{\hbar }^{2}}{2M}{\sigma }_{{K}_{X}}^{2}=\frac{\pi {\hbar }^{2}}{M}\frac{1}{{S}_{coh}}$$

Here, we replaced ${\sigma }_{{K}_{X}}$ by 1/2*σ*_*X*_ and we applied the Gaussian wave packet to identify *σ*_*X*_ with *σ*. A difficulty to use the above equation is the uncertainty on the hole effective mass for CdSe, which is highly anisotropic and reported values range from 0.45 to 1.21 along [100] and from 1.61 to 1.92 along [111]. Even so, taking the total exciton mass *M* = *m*_*e*_ + *m*_*h*_ equal to the free electron mass, we obtain a localization energy of ≈35 meV; a figure that may overestimate Δ*E* but is still comparable to thermal energy at room temperature.

**Fig. 7:**
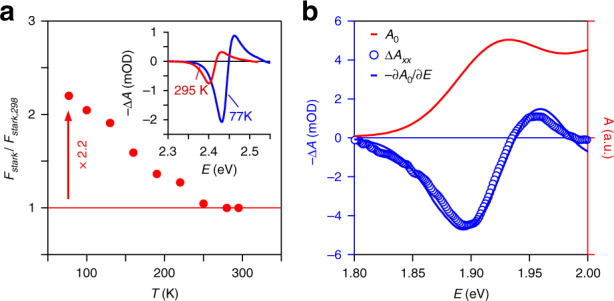
Temperature dependent and QD OSE. **a** Variation of *F*_Stark_ as a function of temperature, as measured on 4.5 ML CdSe nanoplatelets. The inset shows the instantaneous transient absorbance as recorded at (red) 295 K and (blue) 77 K. **b** Measurement of the optical Stark effect on a film of 6.4 nm zinc-blende CdSe quantum dots at room temperature, including (red line) the absorption spectrum *A*_0_ of the quantum dots, (blue markers) the instantaneous transient absorbance, and (blue line) the energy derivative of the absorption spectrum rescaled to highlight the agreement with the transient absorbance spectrum

The correspondence between the localization energy and thermal energy suggests that exciton localization is in part caused by thermal effects. To assess this point, we analyzed the variation of *F*_*Stark*_ as a function of temperature, down to 77 K, see Supporting Information S8 for experimental details. As can be seen in Figure 7a, the instantaneous transient absorbance when pumping below the band-gap corresponds to the derivative of the absorption spectrum at 295 K and 77 K, alike. However, reducing temperature to 77 K significantly enhances the magnitude of the transient absorbance under similar pump conditions. While a quantification of this transient absorbance into an energy shift Δ*E* requires the absorbance spectrum and the screening factors *f*_*xx*_ and *f*_*xy*_ at the relevant temperature, a first estimate of Δ*E* can be obtained by assuming these quantities to be temperature independent. This is not unreasonable as the shape of the transient absorption spectrum measured at 77 K matches quite well the derivative of the absorption spectrum measured at 295 K. As shown in Figure 7a, such an analysis leads to an estimated increase of *F*_*Stark*_ by more than a factor of 2 when cooling down the nanoplatelets to 77 K. We thus conclude that thermal effects directly contribute to exciton localization.

When measuring the optical Stark effect through sub-bandgap pump-probe spectroscopy, the pump pulse does not created excitons but rather changes the photon energy at which excitons are formed. Exciton localization by thermal effects can therefore involve temperature-dependent changes of the potential energy landscape, for example linked to lattice vibrations, not an activation energy that may prevent initially formed excitons from exploring a static the potential energy landscape at low temperature. In the absence of any additional localization due to static imperfections, lowering the temperature could thus lead to fully delocalized excitons under cryogenic conditions, as argued by Naeem et al.^14^ through four-wave mixing experiments at 4 K. Under such conditions, *F*_*Stark*_ will increase to become equal to *F*_*Abs*_, and the radiative lifetime of the bright exciton will shorten by a factor *S*_*coh*,298_ K/*S*. In the case of 326 nm^2^ nanplatelets, the resulting *τ*_*rad*,4 K_ would amount to 105 ps, a number that agrees with published experimental results^1,14^.

Acknowledging that excitons in 2D nanoplatelets exhibit a significant localization of the exciton center-of-mass, the question arises as to how this compares to 0D quantum dots. To address this point, we analyzed the non-linear Stark effect of a film consisting of 6.25 nm CdSe quantum dots. For details on the synthesis and properties of the QDs, see Methods section and Supporting Information S1. Fig. 7b represents the absorbance spectrum and a Δ*A* trace at zero time delay recorded on this film upon excitation using a 700 nm pump laser, see Supporting Information S10 for more details. Also in this case, it can be seen that this pulse, which has a detuning Δ of 150 meV relative to the band-edge transition, induces a transient absorbance around the band-edge that resembles the derivative of the absorbance spectrum. From the corresponding energy shift, we estimate an oscillator strength *F*_*Stark*_ = 8.4 for the band-edge exciton. On the other hand, calculating the oscillator strength from the integrated absorption coefficient spectrum yields *F*_*abs*_ = 11.9, see Supporting Information S10. This number agrees with published values^38^, and highlights that, opposite from CdSe nanoplatelets, the ratio *F*_*Stark*_/*F*_*abs*_ is around 1 in the case of CdSe quantum dots. Hence, for 0D quantum dots, the coherence volume of the exciton and the volume of the nanocrystal effectively coincide, i.e., the exciton is fully delocalized over the entire nanocrystal. Clearly, this lack of localization is intrinsically linked to the electron and the hole occupying states showing 3-dimensional quantization with quantization energies exceeding the localization energy. Hence, one could use the finding that the ratio *F*_*Stark*_/*F*_*abs*_ is near-unity as a fingerprint of strong confinement and thus identify zero-dimensional quantum systems.

In conclusion, we analyzed the oscillator strength of the exciton transition in 4.5 monolayer CdSe nanoplatelets. By means of the optical Stark effect, induced and measured through contact-free femtosecond pump-probe spectroscopy, we obtain an oscillator strength that is smaller by a factor of 50 than the oscillator strength derived from the integrated absorption coefficient. We attribute this difference to exciton localization within the CdSe nanoplatelet. Since multiple localized excitons can be formed, localization does not affect the absorption coefficient of the exciton transition. However, since coherence between different localized excitons is lost within the time span of the *ca*. 100 femtosecond pump pulse, the Stark shift of individual localized excitons is measured^35^. Having rationalized the ratio between both oscillator strengths as the ratio between the exciton coherence area and the total nanoplatelet area, we obtain an exciton coherence area of 6.1 nm^2^ at room temperature. Importantly, opposed to commonly used state-filling models, we can use the optical Stark measurement to calculate the exact radiative lifetime, without any assumptions^19,20^. This internally consistent picture indicates that the coherence area of excitons in 4.5 ML CdSe nanoplatelets is considerably smaller than the total nanoplatelet area.

When reducing temperatures to 77 K, the coherence area increases more than twofold, suggesting that exciton localization is at least partially a thermal effect. This conclusion is supported by the fact that thermal energy at room temperature can suffice to localize the exciton center-of-mass in the observed coherence area. Remaining decoherence and localization will most likely stem from crystal imperfections such as twin defects, missing surface ligands, etc. Clearly, the expected giant oscillator strength does not manifest itself at room temperature due to intrinsic limitations of the material, rather than impurity or defect localization, thereby limiting the potential for their use in strong light-matter coupling scenarios at room temperature. When applying the same approach to CdSe QDs, we find that oscillator strengths measured through the optical Stark effect and the integrated absorption coefficient are comparable. This suggests that in such systems, electron-hole pairs are fully delocalized across the entire quantum dot volume and that the agreement between both oscillator strengths can be used as a descriptor to identify zero-dimensional quantum systems. Finally, our work suggests that the numerous reports on transition dipole moments in 2D materials report the oscillator strength of strongly localized excitons. The commonly used OSE experiment is as such more of a probe for exciton localization and the effective oscillator strength under the measurement conditions, rather than revealing the maximum achievable oscillator strength, relevant for device applications.

## Materials and methods

### Synthetic methods

#### Chemicals

Toluene (>99.8%), methanol (>99.85%), isopropanol (>99.7%) and acetone (>99.5%) were purchased from Fiers; oleic acid (90%), cadmium oxide (>99.99% metals bases), selenium (99.999%) and 1-octadecene (ODE, tech.) were purchased from Alfa Aesar; trioctylphospine (TOP, 97%) was purchased from Strem Chemicals. All chemicals were used without further purification.

#### Synthesis

Details on the synthesis of varying area nanoplatelets and bulk-like CdSe quantum dots is laid out in the Supplementary Information.

### Pump-probe spectroscopy

#### Setup

Samples were excited using 120 femtosecond pump pulses with varying wavelengths created from the 800 nm fundamental (Spitfire Ace, Spectra Physics) through non-linear conversion in an OPA (Light Conversion TOPAS). Equally short probe pulses were generated in a 2 mm CaF_2_ crystal using the 800 nm fundamental. The pulses were delayed relative to the pump using a delay stage with 33 fs bi-directional accuracy. The probe spectrum in our experiments covers the UV-VIS window from 350 nm up to 750 nm, yet we focus our attention on the region near the band edge, i.e., the heavy-hole transition at 510 nm for the CdSe platelets and the 1S-1S transitions manifold at 645 nm for the CdSe QDs. Pump and probe pulses were linearly polarized using appropriate polarization optics, in particular a broadband quartz-MgF_2_ quarter wave plate (Newport) is used for the probe and a Bérek compensator or calcite polarizer (Newport) is used to rotate or fix the pump polarization. For variable temperature experiments, the same samples as for the room temperature experiments are loaded in a vacuum contact cryostat which is backfilled with liquid nitrogen.

#### Photon flux calculation

The photon flux is calculated from the average power, the repetition rate and the beam area. The latter is obtained through a Thorlabs CCD beam profiler, and defined as *A*_*beam*_ = 2*π* × *σ*_*x*_*σ*_*y*_ where *σ*_*i*_ is the standard deviation in the *i* = *x*, *y* direction.

## Supplementary information

Supplementary Information
