# Supplementary material for: Localization-limited exciton oscillator strength in colloidal CdSe nanoplatelets revealed by the optically induced stark effect
Source: Light Sci Appl. 2021 May 31;10:112. doi: 10.1038/s41377-021-00548-z (PMC8165098; doi:10.1038/s41377-021-00548-z)
Supplement: Supplementary file 1 — Supplementary Information [file 41377_2021_548_MOESM1_ESM.pdf]

Localization-Limited Exciton Oscillator Strength in Colloidal CdSe Nanoplatelets Revealed by The Optically Induced Stark Effect - Supporting Information

Pieter Geiregat,[*,†,‡] Carmelita Rodà,[†,‡] Ivo Tanghe,[†,¶,‡] Shalini Singh,[§] Alessio Di Giacomo,[†] Delphine Lebrun,[†] Gianluca Grimaldi,[‖] Jorick Maes,[†,‡] Dries Van Thourhout,[¶,‡] Arjan J. Houtepen,[⊥] and Zeger Hens[*,†,‡]

†*Physics and Chemistry of Nanostructures, Department of Chemistry, Ghent University, Belgium*

‡*Center for Nano and Biophotonics, Ghent University, Belgium*

¶*Photonics Research Group, Ghent University, Belgium*

§*Department of Chemical Sciences and Bernal Institute, University of Limerick, Limerick Ireland*

‖*Center for Nanophotonics, NWO-Institute AMOLF, Science Park 104, 1098 XG Amsterdam, The Netherlands*

⊥*Opto-Electronic Materials Section, Department of Chemical Engineering, Delft University, Delft, The Netherlands*

E-mail: pieter.geiregat@ugent.be; zeger.hens@ugent.be

Contents

S1 Further Material Characterization **S4**

S1.1 Synthesis of different aspect ratio 4.5 ML CdSe Nanoplatelets S4

S1.2 Sizing of 4.5 ML CdSe Nanoplatelets S5

S1.3 Characterization of CdSe Quantum Dots S6

S1.4 Sample Treatment and Conditions S6

S2 Linear Absorption and Oscillator strength **S8**

S2.1 Intrinsic Absorption Coefficient of CdSe Platelets S8

S2.2 Quantum Well Absorption Spectrum S10

S2.3 Oscillator Strength of Platelets S11

S3 Radiative Lifetime of CdSe Nanoplatelets **S14**

S4 Occurrence of Two-Photon Absorption **S15**

S5 Polarization Analysis **S16**

S6 Dipole Moment and Rabi Splitting from Stark Measurements **S18**

S7 Exciton Localization and Oscillator Strength **S19**

S7.1 Electron-Hole Pair States as a Basis S19

S7.2 The Exciton Wavefunction S20

S7.3 The Matrix Element for Interband Optical Transitions S21

S7.4 Transitions to Delocalized, Bound Excitons S23

S7.5 Transitions to Localized, Bound Excitons S24

S7.6 The Coherence Area of a Gaussian Wavepacket S25

S8 Low Temperature Measurements **S26**

S9 Nanoplatelets with Different Surface Area **S27**

S10 Stark Measurements on CdSe Quantum Dots **S28**

S10.1 Synthesis of Weakly Confined CdSe Quantum Dots. S28

S10.2 Intrinsic Absorption Coefficient of CdSe Quantum Dots S29

S10.3 Oscillator Strength S29

S10.4 OSE Spectroscopy S30

References **S31**

S1 Further Material Characterization

S1.1 Synthesis of different aspect ratio 4.5 ML CdSe Nanoplatelets

The synthesis of 4.5 ML NPLs with different aspect ratios was slightly adapted from reported procedures. Briefly, in a 25 mL three-neck round-bottom flask, 0.30 mmol of Cd(myristate)2 and 0.30 mmol of Se powder were mixed with 12 mL of ODE. The suspension was degassed for 1h at 100 $°C$ then, under N2 atmosphere, the temperature was set to 240 $°C$ and when the suspension became deep orange (at 220 $°C$), 0.6 mmol of Cd(ac)2 or Cd(ac)2·2H2O were swiftly injected. The reaction temperature was set at 240 $°C$, and the time was varied from 8 to 10 minutes to tune the lateral dimensions. Afterward, the suspension was cooled and when the temperature reached 160 $°C$, 2 mL of OA were injected, followed by cooling to 80 $°C$. The crude synthesis product was mixed with 20 mL of n-hexane, and 15 mL of a 1:5(v/v) solution of IPA/EtOH was added. the suspension was centrifuged for 10 min at 4300 rpm. The NPLs precipitated under these conditions, while the byproducts (dots, clusters) remained in the liquid phase. The supernatant was discarded, and the solid was redispersed in n-hexane and centrifuged at 4200 rpm to remove the unreacted carboxylates. The solid was discarded, and the supernatant, containing CdSe NPLs, was collected in and stored as n-hexane dispersion. Sample AD93 was synthetized in a similar fashion, but without using the Schlenk apparatus. Briefly, in a 20 mL vial, 0.30 mmol of 0.30 mmol of Cd(myristate)2 and 0.30 mmol of Se powder were mixed with 12 mL of ODE. Under vigorous stirring, on a heating plate the temperature was to 240 $°C$ and, at 220 $°C$ (deep orange dispersion) 0.6 mmol of Cd(ac)2·2H2O were swiftly injected. The reaction time was 9 minutes at 240 $°C$ and then 2 mL of OA was injected. The purification procedure was exactly the same as the one mentioned for the NPLs synthetized using the Schlenk apparatus.

S1.2 Sizing of 4.5 ML CdSe Nanoplatelets

Transmission electron microscopy images of the 4.5 ML CdSe nanoplatelets used in this work are shown in Figure 1 of the main paper. The main analysis was done on this sample with average dimensions of $34 \times 9.6\,\text{nm}^2$ ($= 326$ nm^2). The histograms of this nanoplatelet's length and width are shown in Figure 1 below. Figure 2 shows TEM images of the other lateral areas used in this work, varying from 65 (20 x 3.4 nm^2) to 180 nm ^{2} (46.2 x 4.0 nm^2).

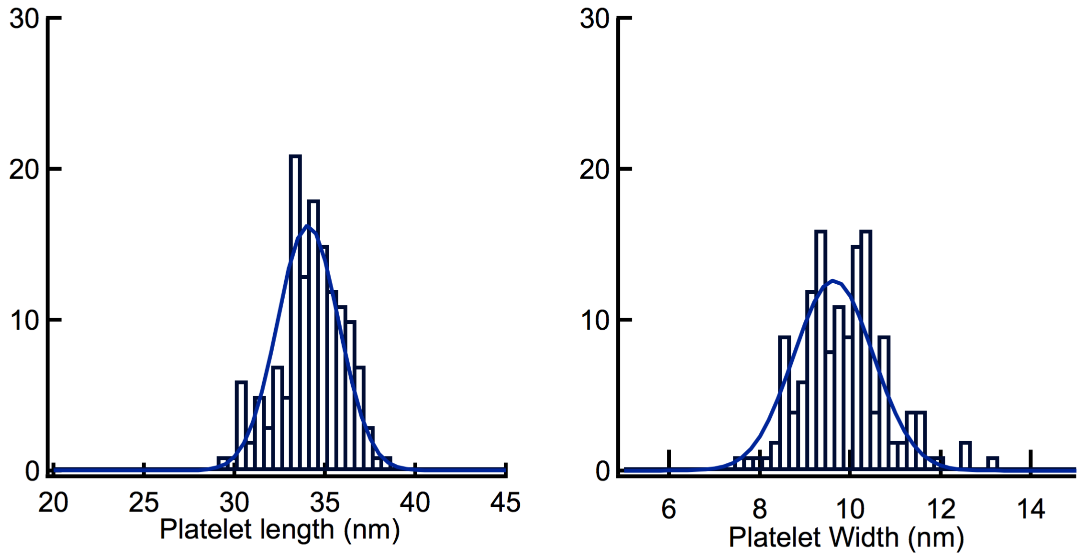

Figure S1: Transmission electron microscopy analysis of the lateral dimensions of the largest area 4.5 ML nanoplatelet sample used in this work.

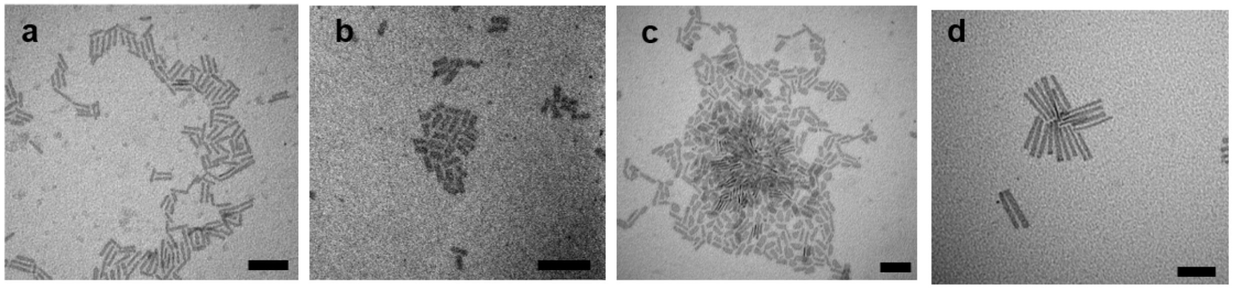

Figure S2: TEM images of smaller 4.5 ML CdSe samples used in this work: (a) 68 nm^2, (b) 106 nm^2, (c) 125 nm^2, (d) 180 nm^2. All scale bars are 50 nm.

S1.3 Characterization of CdSe Quantum Dots

Figure S3 displays a bright-field TEM image of the CdSe QDs used in this study, together with an absorbance spectrum. The band-edge absorbance peaks at a photon energy of 1.93 eV, from which we estimate an average QD diameter of 6.25 nm using the sizing curve published by Maes *et al.*.[1]

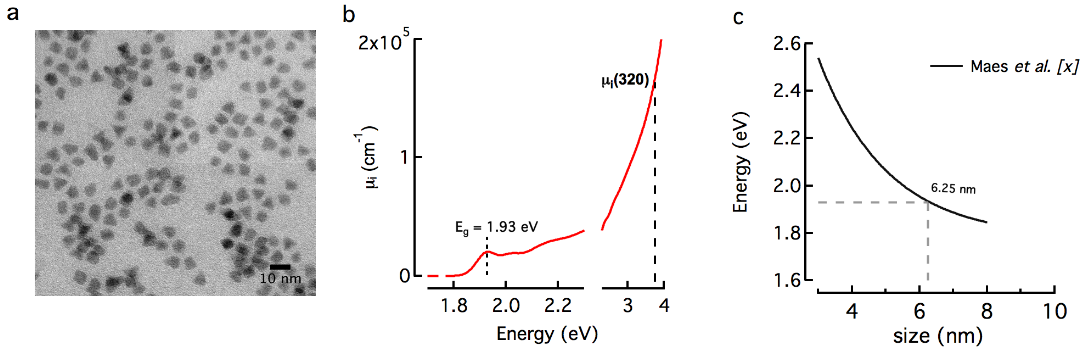

Figure S3: CdSe quantum dots (a) Transmission electron microscopy imaging of the 6.25 nm CdSe quantum dots used in this work. (b) Absorption spectrum normalized to represent μ_i. Indicated are the band gap (1.93 eV) and the normalization point at 320 nm (3.87 eV) (c) Sizing curve of Maes *et al.* used to determine the size of 6.25 nm.[1]

S1.4 Sample Treatment and Conditions

The samples were synthesized in air-free conditions and stored in a glovebox and in the dark. For the measurements, fresh thin films were spincoated on glass substrates in ambient conditions and the experiments were also carried out in ambient conditions. As is well-known, experiments on nanoplatelets relying on generation of real charges (luminescence, gain, photodetection, ...) will be affected by effects such as oxidation or charging. However, Stark measurements explicitly do not produce any real charges in the material. By pumping below the band gap transition, we merely rely on an electric-field induced shift of the absorption line. As no real charges are generated, no effects as charging can occur. Finally, we should note that the experiments at variable temperature (including room temperature), were carried out under vacuum conditions in the cryostat chamber. We obtained similar values for

Fstark and similar dynamics as under ambient conditions.

S2 Linear Absorption and Oscillator strength

S2.1 Intrinsic Absorption Coefficient of CdSe Platelets

Experimentally, the intrinsic absorption coefficient μ_i of the platelets can be determined by measuring the absorbance in a cuvette and elemental analysis. Indeed $\mu_i = \frac{\ln(10)A}{f_V \cdot L}$, where A is the absorbance, f_V is the volume fraction of the platelets and L is the length of the cuvette. However, as was shown by Achtstein *et al.* , it is also valid to calculate μ_i experimentally, especially in a region where there is no difference between the intrinsic absorption of platelets and bulk material. Here, we choose to do this at 300 nm, similar to Achtstein *et al.*.[2]

To do this calculation, Lorentz local field theory is used in combination with the Maxwell-Garnett effective medium approach. If the platelets are considered as oblate ellipsoids and assuming random orientation, the intrinsic absorption can be calculated as follows:

$$\mu_i(\lambda) = \frac{4\pi}{3\lambda n_s(\lambda)} \left(|f_x(\lambda)|^2 + |f_y(\lambda)|^2 + |f_z(\lambda)|^2\right) \cdot n_s(\lambda) \cdot k_s(\lambda) \tag{S1}$$

where the local field factors are defined as follows $i = (x, y, z)$:

$$f_i(\lambda) = \frac{\epsilon_s(\lambda)}{\epsilon_s(\lambda) + L_i(\epsilon_s(\lambda) - \epsilon_{CdSe}(\lambda))} \rightarrow |f_i(\lambda)|^2 = \frac{\epsilon_s^2(\lambda)}{(\epsilon_s(\lambda) + L_i \cdot (\epsilon_{R,CdSe}(\lambda) - \epsilon_S(\lambda))^2 + (L_i \cdot \epsilon_{I,CdSe})^2} \tag{S2}$$

where ϵ_s is the solvent permittivity and ϵ_{CdSe} is the (complex) permittivity of Cdse nanoplatelets. Using the assumption of oblate ellipsoids, the depolarization factors L_i can be calculated evaluating the following integral

$$L_z = \int_0^{+\infty} \frac{xyz}{2(s+z^2)^{3/2}(s+x^2)^{1/2}(s+y^2)^{1/2}} ds \tag{S3}$$

with x, y, z the half-lengths of the ellipsoid. To calculate the other polarization factors L_x and L_y, x, y and z need to be cyclically permuted.

The intrinsic absorption μ_i can now be determined at 300 nm by using the platelet dimensions ($34 \times 9.5 \times 1.21$ nm^3), $\epsilon_{CdSe} = 7.4 + 8.2i$, $\epsilon_s = 1.974$ (n-hexane) and $n_s = 1.405$. Note that we use the bulk dielectric function of CdSe, which at 300 nm is validated previously.[2] These numbers lead to $(L_x, L_y, L_z) = (0.02, 0.12, 0.86)$ and $(|f_x|, |f_y|, |f_z|) = (0.71, 0.95, 0.2)$. From these values, we find:

$$\mu_i(300\text{nm}) = 5.9 \times 10^5\text{cm}^{-1} \tag{S4}$$

Using this value, the whole experimental absorption spectrum can be rescaled since:

$$\mu_i(\lambda) = A(\lambda) \cdot \frac{\mu_i(300)}{A(300)}. \tag{S5}$$

Figure S3a shows the full $\mu_i(\lambda)$ spectrum.

S2.2 Quantum Well Absorption Spectrum

The following quantum-well absorption model is used to model the intrinsic absorption spectrum, see Figure 2):[3,4]

$$p(E) = p_X(E) + \frac{A_C}{2}\left[\mathrm{erf}\left(\frac{(E-E_0)-E_b^x}{\gamma_C}\right)+1\right] \tag{S6}$$

with p_X is the absorption line shape of a quantum well exciton with asymmetric broadening η:

$$p_X(E) = \frac{1}{2\eta}\left[\mathrm{erf}\left(\frac{E-E_0}{\gamma}-\frac{\gamma}{2\eta}\right)+1\right]\exp\left(\frac{\gamma^2}{4\eta^2}-\frac{E-E_0}{\eta}\right) \tag{S7}$$

Here, E_0 and E_b^x are the absolute exciton energy and exciton binding energy, respectively. Further, γ is the linewidth of the absorption peak, and the continuum edge has a step height of A_C and a width of γ_C.

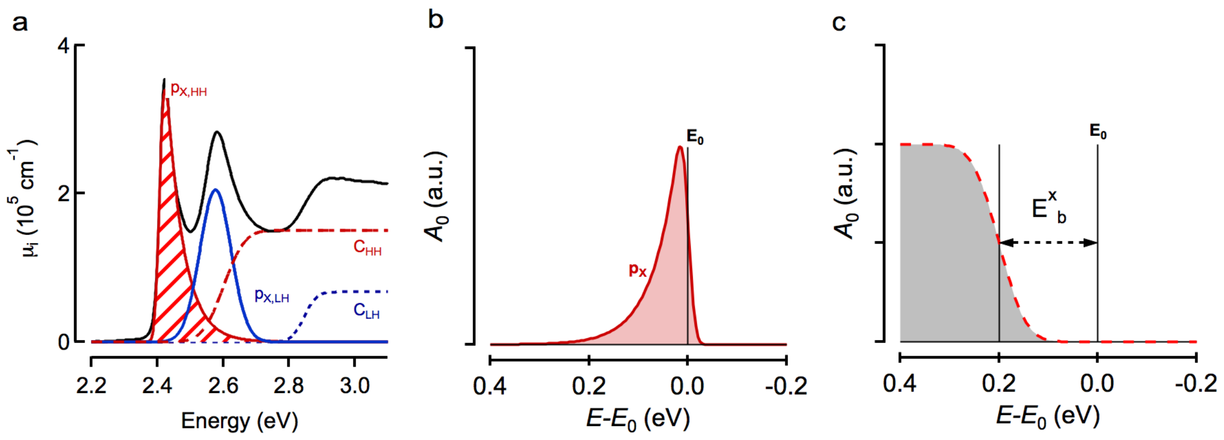

Figure S4: Decomposition of the linear absorption spectrum of the largest area 326 nm^2 4.5ML CdSe sample. (a) Full fit using exicton p and single particle continuum C functions. (b) Zoom on the $p_{X,HH}$ function and the small offset with the zero energy E_0. (c) Definition of the exciton binding energy as the energy seperation between E_0 and the half max position of the continuum step (dashed red).

The total absorption α therefore becomes, including both light (LH) and heavy-hole (HH) contributions:

$$\alpha(E) = A_{HH}\cdot p_{HH}(E) + A_{LH}\cdot p_{LH}(E) \tag{S8}$$

with A_{HH} and A_{LH} the weight of the heavy- and light bands, respectively. A best fit is displayed in Table 1.

The binding energy of 193 meV can be translated into a Bohr radius as:

$$a_B = \frac{\hbar}{\sqrt{2m_r E_b^x}} \tag{S9}$$

Using $m_r = 0.085$, we find $a_B = 1.5$ nm for the 4.5 ML CdSe nanoplatelets used here.[4]

	HH	LH
E_b^x (meV)	193 ± 2	277 ± 0.65
E_0 (meV)	2408 ± 0.05	2568 ± 0.018
γ (meV)	15.94 ± 0.117	65.5 ± 0.001
A_C (meV)	5722 ± 24	2598 ± 40
γ_C (meV)	66.6 ± 2	43.0 ± 1.2
η (meV)	51.3 ± 0.4	9.1 ± 0.001
A	21394 ± 77.3	20976 ± 336

S2.3 Oscillator Strength of Platelets

Knowing the intrinsic absorption of the heavy hole exciton, the oscillator strength of the band gap can be calculated by integrating the heavy hole transition:[5]

$$\mu_{i,gap} = \int_0^{+\infty} p_{X,HH}(E)dE \tag{S10}$$

This integral can be directly used to calculate the oscillator strength:[5]

$$F_{Abs} = \frac{2\epsilon_0 n_s c m_e}{e\pi\hbar}\frac{V_{pl}}{|f_{LF}|^2}\mu_{i,gap} \tag{S11}$$

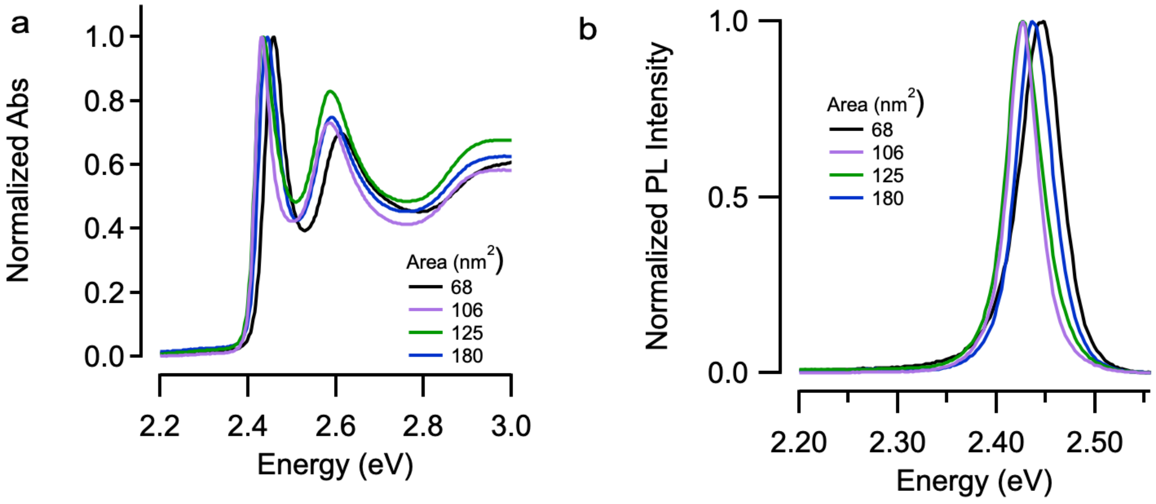

Figure S5: (a) Linear absorption spectrum and (b) luminescence spectrum of smaller area nanoplatelets used in this work.

where the local field factors are calculated in the same way as in section S2, but now around the band gap.

Takingg the case of the largest area sample (326 nm^2), we obtain local field factors (f_x, f_y, f_z) of (0.47,0.87,0.28), which leads to an average local field factor: $|f_{LF}|^2 = 1/3 \times (f_x^2 + f_y^2 + f_z^2) = 0.328$. The nanoplatelet volume is obtained from the TEM analysis as $1.37 \times 34 \times 9.5$ nm^3. The solvent refractive index is taken from hexane, $n_s = 1.5$. Using the fitting values as shown in Table 1, we can reconstruct the function $p_{X,HH}$ which is then integrated on an energy scale. Finally, the oscillator strength F_{Abs} of the heavy hole exciton is obtained:

$$F_{Abs,HH} = 165 \pm 1.3 \tag{S12}$$

The table below lists the calculations for all the nanoplatelet areas used in this work. Note that $F_{abs,HH}$ scales linearly with the nanoplatelet area.

Area (nm^2)	f_x^2	f_y^2	f_z^2	$\lvert f_{LF}\rvert^2$	$F_{Abs,HH}$
326	0.75	0.22	0.09	0.35	165
180	0.96	0.30	0.08	0.45	82
125	0.84	0.42	0.08	0.44	48
106	0.76	0.47	0.07	0.43	42
68	0.87	0.28	0.09	0.41	34

S3 Radiative Lifetime of CdSe Nanoplatelets

Figure S6 shows the fluorescence decay of the 4.5 ML CdSe nanoplatelets dispersed in n-hexane after photo-excitation with a 400 nm pulsed laser diode at 2 MHz. We fit the decay of the integrated PL intensity with a triple exponential decay, similar to Leemans *et al.*:[6]

$$A = A_0 + A_1 e^{-\frac{t}{\tau_1}} + A_2 e^{-\frac{t}{\tau_2}} + A_3 e^{-\frac{t}{\tau_3}} \tag{S13}$$

Similar to their work, we assign the fast component τ_1 to radiative decay, which amounts to 65 percent of the total decay, a number line with the quantum yield of the sample indicating a heterogeneous population of emitters. Given the more complex decay pattern in our core-only nanoplatelets, we decided to assign an average lifetime as:

$$\tau_{av} = \frac{A_1\tau_1 + A_2\tau_2 + A_3\tau_3}{A_1 + A_2 + A_3} = 6.6\text{ns} \tag{S14}$$

Area	326	180	125	106	68
A_1	0.65	0.9	0.83	0.7	0.68
τ_1 (ns)	3.8	3.8	3.6	2.4	1.6
A_2	0.43	0.13	0.18	0.28	0.27
τ_2 (ns)	8.5	15	13	8.8	8.8
A_3	0.04	0.01	0.02	0.03	0.06
τ_3 (ns)	44	76	65	40	48.1
y_0	0.0015	2.5e-4	4.5e-4	5.4e-4	2.1e-3
τ_{PL}	6.6	6	6.4	5.4	6.4

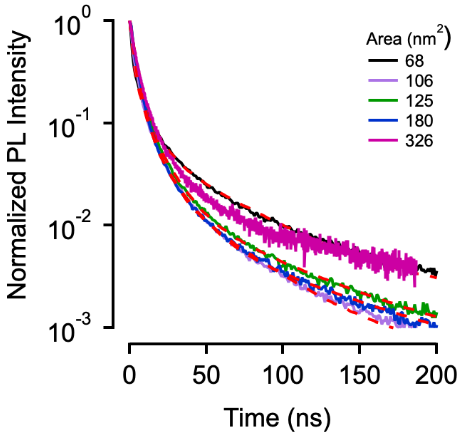

Figure S6: Fluorescence decay after photo-excitation at 400 nm with a 2 MHz pulse train.

S4 Occurrence of Two-Photon Absorption

Upon strong sub-band gap excitation, also real carriers can be generated through 2-photon absorption (2PA).[7] After a 2PA event, a carrier pair is created with a large excess energy. For example, 2PA from a 580 nm pump would create a carrier pair equivalent to a 4.3 eV excitation. We observe such effects at higher pump power as evidenced in Figure S7 where a clear long-lived bleach associated with the creation of real electron-hole pairs is obtained at high pump fluence.

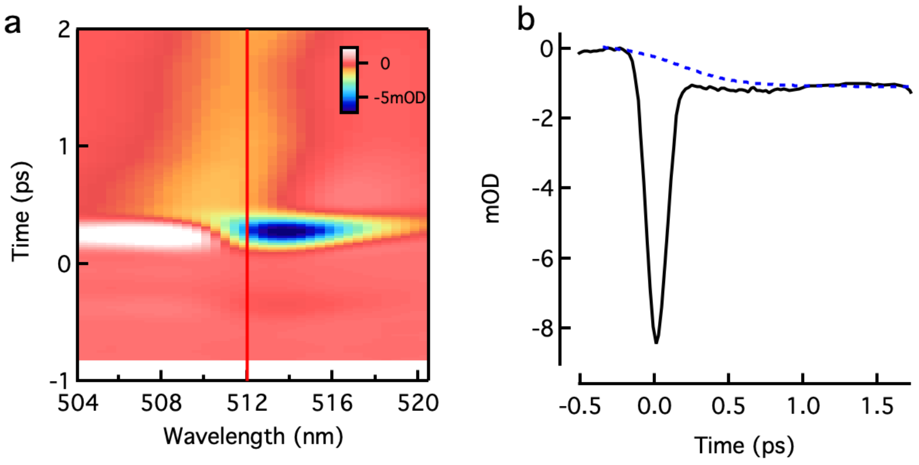

Figure S7: (a) Solvent artefacts by pumping hexane at 580 nm. (b) Effect of 2-photon absorption. Inset shows the scaling of the induced long-lived bleach with pump power.

S5 Polarization Analysis

If we assume a random orientation of the nano-platelets, we can write the absorbance of the thin film A in general as:

$$A = \frac{1}{3}(f_x^2 + f_y^2 + f_z^2)A_0 \tag{S15}$$

where f_i is here taken as the absolute value of the local field factor, as defined in section S2. We note that it needs to be evaluated at the position where the OSE is evaluated (514 nm). The absorbance A_0 is that of unscreened nanoplatelets.

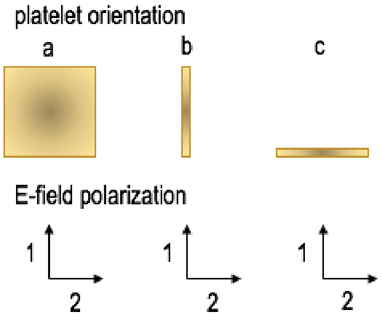

Figure S8: Orientation effect on polarization analysis, overview of notation.

We measure an energy shift ΔE which is an average over all orientations of the nanoplatelets. Given this random orientation of the platelets, we can write:

$$\Delta A_{xx} = \frac{1}{3}\frac{\partial f_x^2 A_0}{E} f_x^2 \Delta E + \frac{1}{3}\frac{\partial f_y^2 A_0}{E} f_y^2 \Delta E + \frac{1}{3}\frac{\partial f_z^2 A_0}{E} f_z^2 \Delta E \tag{S16}$$

$$\Delta A_{xy} = \frac{1}{3}\frac{\partial f_x^2 A_0}{E} f_y^2 \Delta E + \frac{1}{3}\frac{\partial f_x^2 A_0}{E} f_z^2 \Delta E + \frac{1}{3}\frac{\partial f_y^2 A_0}{E} f_z^2 \Delta E \tag{S17}$$

If we replace A_0 for A using the expression above, we obtain:

$$\begin{aligned} \Delta A_{xx} &= \frac{f_x^4 + f_y^4 + f_z^4}{f_x^2 + f_y^2 + f_z^2} \times \frac{\partial A}{\partial E}\Delta E \\ \Delta A_{xy} &= \frac{f_x^2 F_y^2 + f_x^2 f_z^2 + f_y^2 f_z^2}{f_x^2 + f_y^2 + f_z^2} \times \frac{\partial A}{\partial E}\Delta E \end{aligned} \tag{S18}$$

The equations above define the factors f_{xx} and f_{yy} as used in the manuscript:

$$\begin{aligned} f_{xx} &= \frac{f_x^4 + f_y^4 + f_z^4}{f_x^2 + f_y^2 + f_z^2} \\ f_{xy} &= \frac{f_x^2 f_y^2 + f_x^2 f_z^2 + f_y^2 f_z^2}{f_x^2 + f_y^2 + f_z^2} \end{aligned} \tag{S19}$$

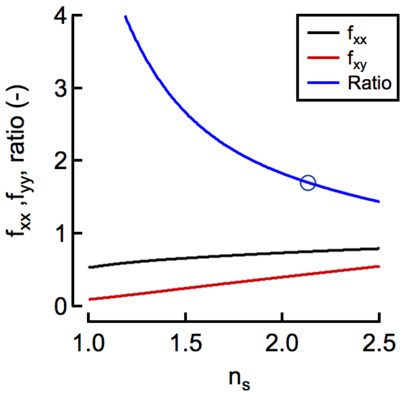

Figure S9: Calculated f_{xx}, f_{xy} and their ratio for varying refractive index of the NPL environment. We obtain a correspondence with the experimental value of 1.7 for a refractive index of 2.13, a reasonable number for a mixture of nano-platelets ($n \approx 3$) and oleate ligands ($n \approx 1.5$).

S6 Dipole Moment and Rabi Splitting from Stark Measurements

Dipole Moment Within the dressed atom picture presented in equation (1) of the main paper, the energy shift ΔE induced by a pump light field detuned by an energy amount $\hbar\Delta\omega$, should be proportional to the light intensity, or square of the average electric field $\langle F \rangle$, and the dipole moment of the transition $\mu_{0\to X}$:

$$\Delta E = \frac{\mu_{0\to X}^2 \langle \mathcal{E} \rangle^2}{\hbar\Delta\omega} \tag{S20}$$

The average electric field $\langle F \rangle$ is linked to the average intensity I_0 through: $\langle \mathcal{E} \rangle^2 = 2I_0/(n\epsilon_0 c)$, where n is the refractive index. ϵ_0 c are the vacuum permittivity and speed of light respectively.[8] The intensity is calculated as $\frac{E_p}{A \times t_p}$ where $t_p = 120$ fs is the pulse width, A is the beam area and E_p is the energy per pulse.

Rabi Splitting and Energy Shifts Apart from the dipole moment, several other quantities are used literature to describe the light-matter coupling strength. These all scale proportional to the dipole moment, but we report here the extracted values for the Rabi splitting energy $\hbar\Omega_0$ and the energy shift ΔE we obtained for the various NPL areas studied under a fluence of 0.6 GW/cm^2.

Area (nm^2)	$\hbar\Omega_0$ (meV)	ΔE (meV)
68	20.75	0.63
106	20.43	0.62
125	17.85	0.48
180	19.88	0.60

S7 Exciton Localization and Oscillator Strength

S7.1 Electron-Hole Pair States as a Basis

To discuss exciton localization and the exciton oscillator strength, we take the approach that states in a crystalline solid can be described as the product of a Bloch wavefunction and an envelope wavefunction, the latter being a solution to the effective mass Schrödinger equation. In that case, the unperturbed single-particle states in the conduction and valence band correspond to free particle waves characterized by wavevectors $\mathbf{k_e}$ and $\mathbf{k_h}$. In coordinate representation, where $\mathbf{r_e}$ and $\mathbf{r_h}$ are the coordinates of the electron and the hole, respectively, the corresponding states can be written as:

$$\langle\, \mathbf{r_e} \,|\, \mathbf{k_e} \,\rangle = \frac{1}{L^{n/2}} \exp\left(i\mathbf{r_e} \cdot \mathbf{k_e}\right) \tag{S21}$$

$$\langle\, \mathbf{r_h} \,|\, \mathbf{k_h} \,\rangle = \frac{1}{L^{n/2}} \exp\left(i\mathbf{r_h} \cdot \mathbf{k_h}\right) \tag{S22}$$

Here, n referst to the dimensionality of the material at hand.

To describe a general electron-hole pair state – such as a bound exciton or a localized exciton – the free particle product states $|\, \mathbf{k_e}, \mathbf{k_h} \,\rangle = |\, \mathbf{k_e} \,\rangle |\, \mathbf{k_h} \,\rangle$ can be taken as a basis. In that case, each electron-hole pair state $|\, X \,\rangle$ can be expanded as:

$$|\, X \,\rangle = \sum_{\mathbf{k_e}, \mathbf{k_h}} \langle\, \mathbf{k_e}, \mathbf{k_h} \,|\, X \,\rangle |\, \mathbf{k_e}, \mathbf{k_h} \,\rangle = \sum_{\mathbf{k_e}, \mathbf{k_h}} \Phi^X(\mathbf{k_e}, \mathbf{k_h}) |\, \mathbf{k_e}, \mathbf{k_h} \,\rangle \tag{S23}$$

Here, the expansion coefficient $\Phi^X(\mathbf{k_e}, \mathbf{k_h})$ is introduced as a mere representation of the inner product $\langle\, \mathbf{k_e}, \mathbf{k_h} \,|\, X \,\rangle$. A similar starting point was put forward by Dresselhaus and by Elliott.[9,10]

To make Eq S23 more tangible, one can re-express the inner product $\langle\, \mathbf{k_e}, \mathbf{k_h} \,|\, X \,\rangle$ by means of the completeness relation in the electron and hole coordinates $\mathbf{r_e}$ and $\mathbf{r_h}$. We thus

obtain:

$$|\,X\,\rangle = \sum_{\mathbf{k_e},\mathbf{k_h}} \left(\int \langle\, \mathbf{k_e}, \mathbf{k_h} \,|\, \mathbf{r_e}, \mathbf{r_h} \,\rangle \langle\, \mathbf{r_e}, \mathbf{r_h} \,|\, X \,\rangle d\mathbf{r_e} d\mathbf{r_h} \right) |\, \mathbf{k_e}, \mathbf{k_h} \,\rangle \tag{S24}$$

Here, the inner product can be made explicit using Eqs S21 and S22, whereas $\langle\, \mathbf{r_e}, \mathbf{r_h} \,|\, X \,\rangle$ is the wavefunction $\Psi^p(\mathbf{r_e}, \mathbf{r_h})$ of the electron-hole pair state $|\, X \,\rangle$. Accordingly, we can rewrite the expansion coefficient $\Phi^X(\mathbf{k_e}, \mathbf{k_h})$ as:

$$\Phi^X(\mathbf{k_e}, \mathbf{k_h}) = \frac{1}{L^n} \int e^{-i(\mathbf{k_e}\cdot\mathbf{r_e}+\mathbf{k_h}\cdot\mathbf{r_h})} \Psi^X(\mathbf{r_e}, \mathbf{r_h}) d\mathbf{r_e} d\mathbf{r_h} \tag{S25}$$

Hence, we retrieve the familiar result that the expansion coefficients of a quantum state $|\, X \,\rangle$ in coordinate and wave vector representation are coupled by a Fourier transform; a result that is well known from free particle quantum mechanics. Using the properties of Fourier transform pairs, we also have:

$$\Psi^X(\mathbf{r_e}, \mathbf{r_h}) = \left(\frac{L}{4\pi^2} \right)^n \int e^{i(\mathbf{k_e}\cdot\mathbf{r_e}+\mathbf{k_h}\cdot\mathbf{r_h})} \Phi^X(\mathbf{k_e}, \mathbf{k_h}) d\mathbf{k_e} d\mathbf{k_h} \tag{S26}$$

S7.2 The Exciton Wavefunction

The exciton state in a perfect crystal is characterized by an envelope wavefunction $\Psi(\mathrm{r_e}, \mathrm{r_h})$ that is an eigenfunction of a hydrogen-like Hamiltonian, which describes the electron-hole pair bound by a screened Coulomb interaction:

$$\left(-\frac{\hbar^2}{2m_e}\nabla_e^2 - \frac{\hbar^2}{2m_h}\nabla_h^2 - \frac{e^2}{4\pi\epsilon\epsilon_0|\mathbf{r_e} - \mathbf{r_h}|} \right) \Psi(\mathbf{r_e}, \mathbf{r_h}) = E\Psi(\mathbf{r_e}, \mathbf{r_h}) \tag{S27}$$

Here, all symbols have their usual meaning.

This Schrödinger equation is conveniently solved by a transformation from the electron and hole coordinates $\mathbf{r_e}$ and $\mathbf{r_h}$ to coordinates $\mathbf{R}$ and $\mathbf{r}$ that describe the center-of-mass of

the electron-hole pair and the electron-hole interdistance:

$$\mathbf{R} = \frac{m_e}{m_e + m_h}\mathbf{r_e} + \frac{m_h}{m_e + m_h}\mathbf{r_h} = \gamma_e\mathbf{r_e} + \gamma_h\mathbf{r_h} \tag{S28}$$

$$\mathbf{r} = \mathbf{r_e} - \mathbf{r_h} \tag{S29}$$

Importantly, the concomitant wavevectors $\mathbf{K}$ and $\mathbf{k}$ read:[1]

$$\mathbf{K} = \mathbf{k_e} + \mathbf{k_h} \tag{S30}$$

$$\mathbf{k} = \gamma_h\mathbf{k_e} - \gamma_e\mathbf{k_h} \tag{S31}$$

It is well known that with this transformation, the solutions $\Psi(\mathbf{r_e}, \mathbf{r_h})$ to the Schrödinger equation are obtained as:[10]

$$\Psi(\mathbf{r_e}, \mathbf{r_h}) = \frac{1}{L^{n/2}} e^{i\mathbf{K}\cdot\mathbf{R}}\chi_\nu(\mathbf{r}) \tag{S32}$$

Here, the center-of-mass motion is described as a plane wave, whereas the internal motion corresponds to a bound or unbound hydrogen-like wavefunction $\chi_\nu(\mathbf{r})$, as characterized by the set of quantum numbers ν.[2]

S7.3 The Matrix Element for Interband Optical Transitions

Following Elliot,[10] we use the expansion of a general electron-hole state $\langle p \rangle$ as expressed by Eq S23 to write the transition matrix element $\langle X \,|\, \mathbf{H}' \,|\, 0 \rangle$ of the light-matter interaction operator $\mathbf{H}' = \boldsymbol{\mathcal{E}} \cdot \boldsymbol{\mu}$ as:[3]

$$\langle X \,|\, \mathbf{H}' \,|\, 0 \rangle = \sum_{\mathbf{k_e},\mathbf{k_h}} \Phi^X(\mathbf{k_e}, \mathbf{k_h}) \langle \mathbf{k_{e,B}} \,|\, \mathbf{H}' \,|\, -\mathbf{k_{h,B}} \rangle \tag{S33}$$

[1]Note that with these expressions, we have $\mathbf{k_e} \cdot \mathbf{r_e} + \mathbf{k_h} \cdot \mathbf{r_h} = \mathbf{K} \cdot \mathbf{R} + \mathbf{k} \cdot \mathbf{r}$

[2]Note that Eq S32 applies to the specific case of a semiconductor with non-degenerate, isotropic band-edges for which electron and hole effective masses are unique scalars. Otherwise, a description of the envelope in terms of the internal coordinate and the average position of the electron-hole pair is more appropriate.

[3]Here, $\boldsymbol{\mathcal{E}}$ is the electric field, whereas $\boldsymbol{\mu} = e\mathbf{r}$ is the dipole operator.

This expression recognizes the fact that the actual transition from the ground state $|\,0\,\rangle$ to the electron-hole pair state $|\,X\,\rangle$ involves the promotion of an electron from the valence band state $|\,-\mathbf{k_h}\,\rangle$ to the conduction band state $|\,\mathbf{k_e}\,\rangle$. Moreover, the subscript B indicates that the Bloch states and not the envelopes must be used for the calculation of the matrix element $\langle\,\mathbf{k_{e,B}}\,|\,\mathbf{H}'\,|\,-\mathbf{k_{h,B}}\,\rangle$.

In coordinate representation, the matrix element $\langle\,\mathbf{k_{e,B}}\,|\,\mathbf{H}'\,|\,-\mathbf{k_{h,B}}\,\rangle$ becomes an integral over the $\mathbf{r_e}$ coordinate:

$$\langle\,\mathbf{k_{e,B}}\,|\,\mathbf{H}'\,|\,-\mathbf{k_{h,B}}\,\rangle = \frac{1}{L^n}\int e^{-i\mathbf{k_e}\mathbf{r_e}}u_{\mathbf{k_e}}\mathbf{H}'e^{-i\mathbf{k_h}\mathbf{r_e}}u_{\mathbf{k_h}}d\mathbf{r_e} \tag{S34}$$

To evaluate this matrix element, we introduce the following ideas:

- For $\mathbf{k_e} = \mathbf{k_h} = \mathbf{0}$, the integrandum will have the full periodicity of the lattice. Hence, the matrix element $\langle\,\mathbf{0}\,|\,\mathbf{H}'\,|\,\mathbf{0}\,\rangle$ can be calculated as an integral over a single unit cell, normalized by the volume of a unit cell. Hence, it is a number independent of the volume of the semiconductor.

- If either of both wavevectors differs from $\mathbf{0}$, the integrandum is the product of a part with the full periodicity of the lattice, and a wavelike part with wavevector $\mathbf{k_e}+\mathbf{k_h}$.[4] When the crystal is sufficiently large, the matrix element will therefore vanish unless $\mathbf{k_e}+\mathbf{k_h} = \mathbf{0}$. This is the well-know rule that only interband transitions that are *vertical* in reciprocal space are allowed.

In what follows, we will assume that the relevant wavevectors involved in the interband transitions are all small, such that the matrix element $\langle\,\mathbf{k_{e,B}}\,|\,\mathbf{H}'\,|\,-\mathbf{k_{e,B}}\,\rangle$, which is weakly dependent on $\mathbf{k_e}$ can be replaced by $\langle\,c_0\,|\,\mathbf{H}'\,|\,v_0\,\rangle$. Here, $|\,c_0\,\rangle$ and $|\,v_0\,\rangle$ represent the conduction and valence-band states at $\mathbf{k} = \mathbf{0}$. Using this substitution, Eq S33 can be rewritten

[4] In principle, the matrix element adds the wavevector of the electromagnetic field as an additional component here, but this we neglect since electron wavevectors will be much larger than photon wavevectors.

as:

$$\langle X \,|\, \mathbf{H}' \,|\, 0 \rangle = \langle c_0 \,|\, \mathbf{H}' \,|\, v_0 \rangle \left(\sum_{\mathbf{k_e}, -\mathbf{k_e}} \Phi^X(\mathbf{k_e}, -\mathbf{k_e}) \right) \tag{S35}$$

S7.4 Transitions to Delocalized, Bound Excitons

With the factorization of $\Psi(\mathbf{r_e}, \mathbf{r_h})$ into a center-of-mass wavefunction and an internal wavefunction, the Fourier transform linking wavefunction and expansion coefficients $\Phi(\mathbf{k_e}, \mathbf{k_h})$ can be rewritten as:

$$\Phi(\mathbf{k_e}, \mathbf{k_h}) = \frac{1}{L^n} \int e^{-i(\mathbf{K}\cdot\mathbf{R} + \mathbf{k}\cdot\mathbf{r})} \psi(\mathbf{R}) \chi_\nu(\mathbf{r}) d\mathbf{R} d\mathbf{r} \tag{S36}$$

Here, $\psi(\mathbf{R})$ represents a general center-of-mass wavefunction. For the case that $\mathbf{K} = \mathbf{0}$, and assuming free-particle motion for the center-of-mass, this yields:

$$\Phi(\mathbf{k_e}, -\mathbf{k_e}) = \frac{1}{L^{n/2}} \int e^{-i\mathbf{k_e}\cdot\mathbf{r}} \chi_\nu(\mathbf{r}) d\mathbf{r} \tag{S37}$$

Reversing this relation by using the properties of the Fourier integral, we also have:[5]

$$\chi_\nu(\mathbf{r}) = \frac{L^{n/2}}{(2\pi)^n} \int e^{i\mathbf{k_e}\cdot\mathbf{r}} \Phi(\mathbf{k_e}, -\mathbf{k_e}) d\mathbf{k_e} \tag{S38}$$

Evaluating χ_ν at $\mathbf{r} = 0$, we thus obtain:

$$\chi_\nu(\mathbf{r} = \mathbf{0}) = \frac{L^{n/2}}{(2\pi)^n} \int \Phi(\mathbf{k_e}, -\mathbf{k_e}) d\mathbf{k_e} \tag{S39}$$

Referring to Eq S35, we can express the summation over $\mathbf{k_e}$ as an integral by considering that a wavevector range $d\mathbf{k_e}$ will contain $(L/2\pi)^n \times d\mathbf{k_e}$ different wavevectors. Hence:

$$\sum_{\mathbf{k_e}, -\mathbf{k_e}} \Phi(\mathbf{k_e}, -\mathbf{k_e}) = \left(\frac{L}{2\pi} \right)^n \int \Phi(\mathbf{k_e}, -\mathbf{k_e}) d\mathbf{k_e} \tag{S40}$$

[5] Note that when $\mathbf{k_h} = -\mathbf{k_e}$, $\mathbf{k} = \mathbf{k_e}$.

As a result, the transition matrix element to form a delocalized exciton in the bright $|\,\Phi_{\mathbf{K=0}}\,\rangle$ state is obtained as:

$$\langle\,\Phi_{\mathbf{K=0}}\,|\,\mathbf{H}'\,|\,0\,\rangle = L^{n/2}\chi_\nu(\mathbf{0})\langle\,c_0\,|\,\mathbf{H}'\,|\,v_0\,\rangle \tag{S41}$$

Accordingly, we obtain the oscillator strength $F_{\mathbf{K=0}}$ for the formation of a 2D delocalized exciton as:[6]

$$F_{\mathbf{K=0}} = \frac{2m\omega}{\hbar}|\langle\,\Phi_{\mathbf{K=0}}\,|\,x\,|\,0\,\rangle|^2 = \frac{2m\omega}{\hbar}|\chi_\nu(\mathbf{0})|^2\langle\,c_0\,|\,x\,|\,v_0\,\rangle S \tag{S42}$$

We thus conclude that the oscillator strength for the formation of delocalized excitons in the $|\,\Phi_{\mathbf{K=0}}\,\rangle$ scales proportionally with the area S of the quantum well.

S7.5 Transitions to Localized, Bound Excitons

Assuming that the state $|\,X\,\rangle$ of a localized, bound exciton can still be described as the direct product of the center-of-mass part and the internal part, Eq S43 yields:

$$\Phi^X(\mathbf{k_e}, -\mathbf{k_e}) = \frac{1}{L^n}\int\psi(\mathbf{R})d\mathbf{R}\int e^{-i\mathbf{k}\cdot\mathbf{r}}\chi_\nu(\mathbf{r})d\mathbf{r} \tag{S43}$$

In the case of a 2D system, the first integral has units of length, and its square is defined as the coherence area S_{coh} of the exciton center-of-mass:

$$S_{coh} = \left|\int\psi(\mathbf{R})d\mathbf{R}\right|^2 \tag{S44}$$

Following the same arguments as used in the previous section, the matrix element to form this more general electron-hole pair state $|\,p\,\rangle$ is obtained as:

$$\langle\,X\,|\,\mathbf{H}'\,|\,0\,\rangle = S_{coh}^{1/2}\chi_\nu(\mathbf{0})\langle\,c_0\,|\,\mathbf{H}'\,|\,v_0\,\rangle \tag{S45}$$

[6] Here, we take the direction of polarization of the light as $\boldsymbol{x}$, m is the free electron mass and ω is the transition energy.

Accordingly, we obtain the oscillator strength for the formation of a 2D localized exciton in the state $|\, p \,\rangle$ as:

$$F_p = \frac{2m\omega}{\hbar} |\langle\, p \,|\, x \,|\, 0 \,\rangle|^2 = \frac{2m\omega}{\hbar} |\chi_\nu(\mathbf{0})|^2 \langle\, c_0 \,|\, x \,|\, v_0 \,\rangle S_{coh} \tag{S46}$$

We thus conclude that the ratio between the oscillator strength to form a localized and a delocalized amounts to the ratio S_{coh}/S between the coherence area and the total area of the quantum well:

$$\frac{F_X}{F_{\mathbf{K}=0}} = \frac{S_{coh}}{S} \tag{S47}$$

S7.6 The Coherence Area of a Gaussian Wavepacket

In the case of a two-dimensional Gaussian Wavepacket, the center-of-mass motion wavefunction $\psi(\boldsymbol{R})$ can be written as:

$$\psi(\mathbf{R}) = \frac{1}{\sqrt{2\pi}\sigma} \exp\left(-\frac{\mathbf{R}^2}{4\sigma^2}\right) \tag{S48}$$

This yields the coherence area as:

$$S_{coh} = \left| \int \psi(\mathbf{R}) d\mathbf{R} \right|^2 = 8\pi\sigma^2 \tag{S49}$$

S8 Low Temperature Measurements

For the low temperature experiments, samples were again spin-coated on transparent glass slides and mounted in a liquid nitrogen cooled cryostat. Temperature was first ramped down to 77K and gradually increased to room temperature. A pump at 700 nm, 600 meV detuning, was used to initiate the OSE.

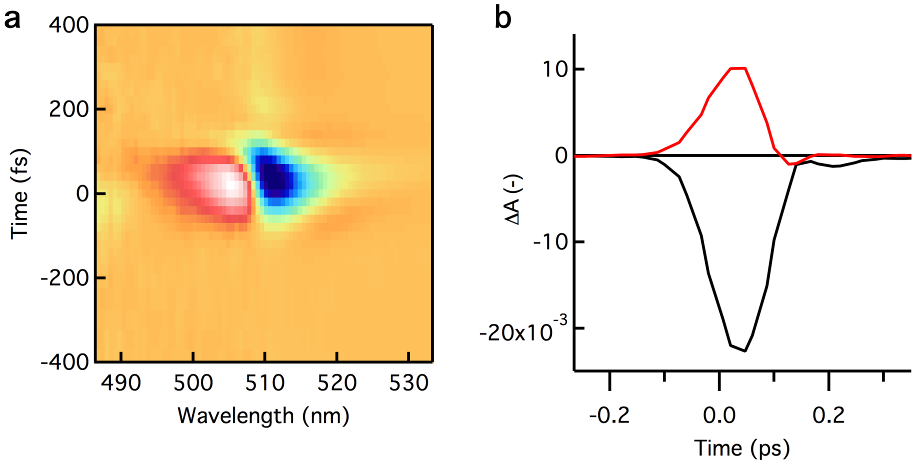

Figure S10: CdSe nanoplatelets at 77K (a) 2D map of ΔA using 700 nm pump. (b) Kinetic traces of (a).

S9 Nanoplatelets with Different Surface Area

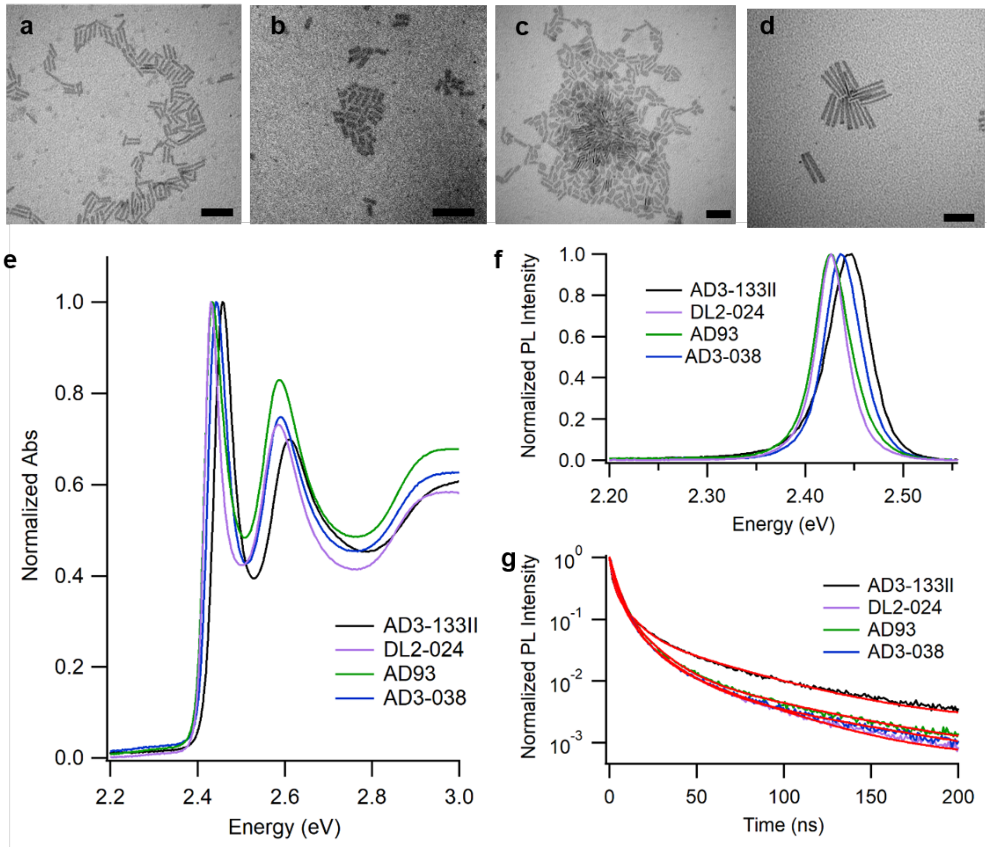

Figure S11: Optical characterization of the investigated 4.5 ML CdSe NPLs. Normalized (e) Absorbance and (d) PL spectra of all investigated samples in hexane solution. The PL spectra were recorded by exciting the samples at 2.64 eV. g) PL-decay curves and multi-exponential fit (red curves) of all investigated samples in hexane solution. The data were fit by using equation (1). The PL-decay curves were recorded by exciting the samples at 2.64 eV with a repletion rate (RR) of 0.5 MHz.

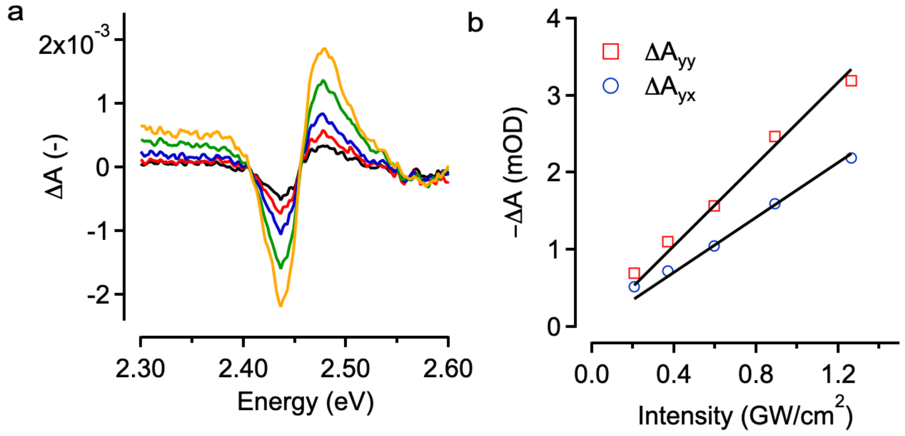

Figure S12: OSE analysis on smallest area nanoplatelets (65 nm^2). (a) ΔA slices at zero delay for increasing pump fluence. (b) Peak ΔA signal for increasing pump fluence for co (xx)- and cross (xy) -polarized experiments.

S10 Stark Measurements on CdSe Quantum Dots

S10.1 Synthesis of Weakly Confined CdSe Quantum Dots.

For the synthesis of CdSe QDs, CdO (0.0128 g, 1 mmol), oleic acid (126 μL, 0.4 mmol) and ODE (8 mL) were mixed and flushed under nitrogen atmosphere at 100 °C for 30 min. The mixture was heated to 245°C to form cadmium oleate and oleylamine (263 μL, 0.8 mmol) was added. To start the reaction, 2 mL of TOPSe (1 M) was added to the mixture. The mixture turns yellow due to the formation of CdSe QDs. After 3 minutes of reaction time, cadmium oleate in ODE (0.1667 M) and TOPSe in ODE (0.1667 M) were continuously added to the synthesis mixture at a rate of 12 mL/h to keep the reaction rate at a high level, which enables to grow large CdSe. The reaction was stopped by thermal quenching using a water bath after approximately 2 h. The dark red reaction mixture was purified by the addition of toluene, isopropanol and methanol, in a 1:1:1 ratio relative to the volume of the reaction mixture. The resulting turbid solution was centrifuged to obtain a precipitate of QDs that was re-dispersed in toluene. Next, purification was repeated three times using toluene and acetone as solvent and non-solvent, respectively, to remove all residual reaction products.

S10.2 Intrinsic Absorption Coefficient of CdSe Quantum Dots

The intrinsic absorption coefficient of spherical zinc blence CdSe QDs can be calculated in a similar manner as for nanoplatelets. The depolarization factors amount to 1/3 in case of a sphere, hence simplifying the formula for μ_i to:

$$\mu_i = \frac{2\pi}{\lambda}\frac{\epsilon_{I,s}}{n_m}|f(\lambda)|^2 \tag{S50}$$

with f the local field factor for a spherical inclusion:

$$f(\lambda) = \frac{3\epsilon_s(\lambda)}{\epsilon_{CdSe}(\lambda) + 2\epsilon_s(\lambda))} \tag{S51}$$

We calculate μ_i at 320 nm, where $|f(320)|^2 = 0.228$, using $\epsilon_{CdSe} = 8 + 6.6i$. This amounts to $\mu_i(320) = 1.65 \times 10^5 \text{cm}^{-1}$. Figure S2b shows the absorption spectrum normalized to represent μ_i.

S10.3 Oscillator Strength

Using the same approach as for the CdSe platelets lined out in S2.3, we can calculate the oscillator strength of the band edge transition in the 6.25 nm CdSe QDs using the integrated intrinsic absorption spectrum:

$$F_{abs} = \frac{2\epsilon_0 n_s c m_e}{e\pi\hbar}\frac{V_{pl}}{|f_{LF}|^2}\mu_{i,gap} \tag{S52}$$

The local field factor is calculated using the expression for spherical inclusions, *i.e.* $L_i = 1/3$ in the framework of the depolarization factors lined out earlier. We obtain a value of: $|f_{LF}|^2 = 0.287$ using bulk dielectric constants of c-CdSe at 1.93 eV, $\epsilon_{1.93eV} = 8 + 1.6i$, and a solvent dielectric constant of 2.25 (toluene, $n_s = 1.5$).[11] $\mu_{i,gap}$ is calculated from the intrinsic absorption spectrum, see Figure S2, by integrating from $+\infty$ to 1.93 eV and doubling the value. We obtain $f_{abs} = 11.9$, in line with the values calculated by Moreels *et al.*[12]

S10.4 OSE Spectroscopy

Figure S13a shows the 2D false colour map of ΔA after a 700 nm pulse (Δ =160 meV). Figure S13b shows a kinetic slice taken at 650 nm. We fit a rising background to accomodate for the creation of real carrier through 2PA (2-photon absorption). Note that this is not required at lower densities where 2PA is negligible.

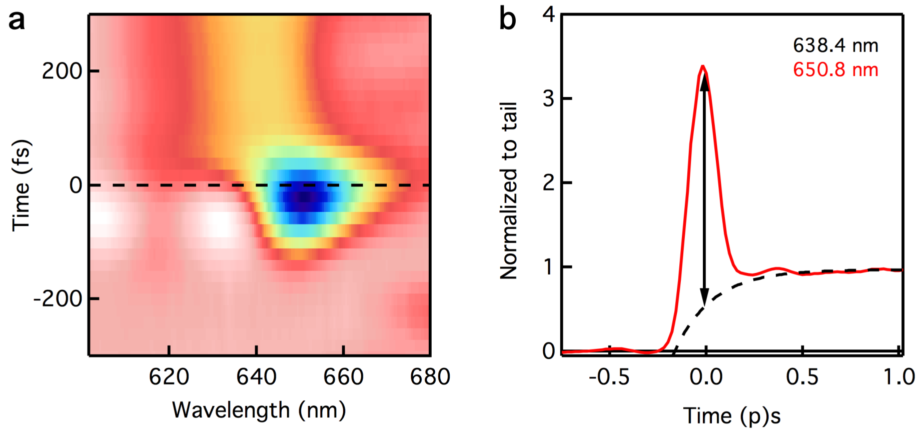

Figure S13: (a) 2D map of ΔA using 700 nm pump. (b) Kinetic trace at 650 nm for extraction of the net Stark contribution.

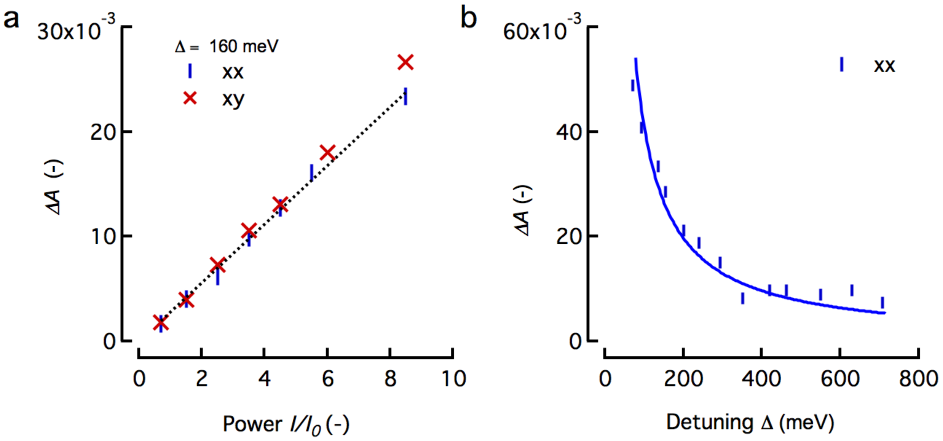

Figure S14: Dressed atom picture for CdSe QDs (a) Sweep of the pump power at fixed detuning (160 meV) and (b) detuning at fixed power ($10I_0$) for the CdSe QD sample.

References

(1) Maes, J.; Castro, N.; De Nolf, K.; Walravens, W.; Abécassis, B.; Hens, Z. Size and Concentration Determination of Colloidal Nanocrystals by Small-Angle X-Ray Scattering. *Chemistry of Materials* **2018**, *30*, 3952–3962.

(2) Achtstein, A. W.; Antanovich, A.; Prudnikau, A.; Scott, R.; Woggon, U.; Artemyev, M. Linear Absorption in CdSe Nanoplates: Thickness and Lateral Size Dependency of the Intrinsic Absorption. *J. Phys. Chem. C* **2015**, *119*, 20156–20161.

(3) Grim, J. Q.; Christodoulou, S.; Di Stasio, F.; Krahne, R.; Cingolani, R.; Manna, L.; Moreels, I. Continuous-wave Biexciton Lasing at Room Temperature using Solution-processed Quantum Wells. *Nat. Nanotech.* **2014**, *9*, 891–895.

(4) Tomar, R.; Kulkarni, A.; Chen, K.; Singh, S.; Thourhout, D. V.; Hodgkiss, J. M.; Siebbeles, L. D. A.; Hens, Z.; Geiregat, P. Charge Carrier Cooling Bottleneck Opens Up Nonexcitonic Gain Mechanisms in Colloidal CdSe Quantum Wells. *J. Phys. Chem. C* **2019**, *123*, 9640–9650.

(5) Hens, Z.; Moreels, I. Light Absorption by Colloidal Semiconductor Quantum Dots. *J. Mater. Chem.* **2012**, *22*, 10406–10415.

(6) Leemans, J.; Singh, S.; Li, C.; Brinck, S. T.; Bals, S.; Infante, I.; Moreels, I. Near-Edge Ligand Stripping and Robust Radiative Exciton Recombination in CdSe/CdS Core/Crown Nanoplatelets. *J. Phys. Chem. Lett.* **2020**, *11*, 3339–3344.

(7) Scott, R.; Achtstein, A. W.; Prudnikau, A.; Antanovich, A.; Christodoulou, S.; Moreels, I.; Artemyev, M.; Woggon, U. Two Photon Absorption in II-VI Semiconductors: The Influence of Dimensionality and Size. *Nano Lett.* **2015**, *15*, 4985–4992.

(8) Yang, Y.; Yang, M.; Zhu, K.; Johnson, J. C.; Berry, J. J.; Lagemaat, J. V. D.;

Beard, M. C. Stark effect in lead iodide perovskites. *Nature Communications* **2016**, *7*, 1–5.

(9) Dresselhaus, G. Effective Mass Approximation for Excitons. *J. Phys. Chem. Sol.* **1956**, *1*, 14–22.

(10) Elliott, R. J. Intensity of Optical Absorption by Excitons. *Phys. Rev.* **1957**, *108*, 1384–1389.

(11) Adachi, S. *Optical Constants of Crystalline and Amorphous Semiconductors*; Springer Science, 1999.

(12) Moreels, I.; Lambert, K.; Muynck, D. D.; Zhao, Q.; Tomme, V.; Vanhaecke, F.; Hens, Z.; Capek, R. K.; Moreels, I.; Lambert, K.; De Muynck, D.; Zhao, Q.; Vantomme, A.; Vanhaecke, F.; Hens, Z. Optical Properties of Zincblende Cadmium Selenide Quantum Dots. *J.Phys. Chem. C* **2010**, *114*, 6371–6376.
